# A unified mechanism for LLPS of ALS/FTLD-causing FUS as well as its modulation by ATP and oligonucleic acids

**DOI:** 10.1371/journal.pbio.3000327

**Published:** 2019-06-12

**Authors:** Jian Kang, Liangzhong Lim, Yimei Lu, Jianxing Song

**Affiliations:** Department of Biological Sciences, Faculty of Science, National University of Singapore, Singapore; University College London Institute of Neurology, UNITED KINGDOM

## Abstract

526-residue Fused in sarcoma (FUS) undergoes liquid–liquid phase separation (LLPS) for its functions, which can further transit into pathological aggregation. ATP and nucleic acids, the universal cellular actors, were shown to modulate LLPS of FUS in a unique manner: enhancement and then dissolution. Currently, the driving force for LLPS of FUS is still under debate, while the mechanism for the modulation remains completely undefined. Here, by NMR and differential interference contrast (DIC) imaging, we characterized conformations, dynamics, and LLPS of FUS and its domains and subsequently their molecular interactions with oligonucleic acids, including one RNA and two single-stranded DNA (ssDNA) molecules, as well as ATP, Adenosine monophosphate (AMP), and adenosine. The results reveal 1) both a prion-like domain (PLD) rich in Tyr but absent of Arg/Lys and a C-terminal domain (CTD) abundant in Arg/Lys fail to phase separate. By contrast, the entire N-terminal domain (NTD) containing the PLD and an Arg-Gly (RG)-rich region efficiently phase separate, indicating that the π-cation interaction is the major driving force; 2) despite manifesting distinctive NMR observations, ATP has been characterized to modulate LLPS by specific binding as oligonucleic acids but with much lower affinity. Our results together establish a unified mechanism in which the π-cation interaction acts as the major driving force for LLPS of FUS and also serves as the target for modulation by ATP and oligonucleic acids through specific binding. This mechanism predicts that a myriad of proteins unrelated to RNA-binding proteins (RBPs) but with Arg/Lys-rich disordered regions could be modulated by ATP and nucleic acids, thus rationalizing the pathological association of Amyotrophic lateral sclerosis (ALS)-causing C9ORF72 dipeptides with any nucleic acids to manifest cytotoxicity.

## Abstract 2

FUS is an RNA-binding protein that is mutated and/or aggregated in several neurodegenerative diseases; this study shows that both ATP and nucleic acids modulate liquid-liquid phase separation of FUS in the same manner by specific binding.

## Introduction

Fused in sarcoma (FUS)/Translocated in Sarcoma (TLS) was first identified as a fusion oncogene in human liposarcomas, which is composed of the FUS residues 1–266 fused with a transcription factor C/EBP Homologous protein (CHOP) [1,2]. FUS is an RNA-binding protein (RBP) belonging to the FUS/TLS, EWS, and TAF15 (FET) protein family, which functions by binding a large array of nucleic acids, including DNA and RNA, to control transcription, RNA processing, cytoplasmic fates of messenger RNAs, and DNA damage responses [3]. On the other hand, a large array of point mutations of the FUS protein were identified to cause neurodegenerative diseases, including Amyotrophic lateral sclerosis (ALS) and frontotemporal lobar dementia (FTLD) [3–5]. FUS is also a key component of cellular granules, including stress granules (SGs) composed of both RBPs and nucleic acids in response to environmental stresses [3–11]. Unlike the classic protein–nucleic acid complexes such as ribosome, SGs are dynamic assemblies behaving as liquid droplets formed through a physicochemical process known as liquid–liquid phase separation (LLPS), which is now recognized to be a common principle for forming various cellular membraneless organelles [12–17]. Intriguingly, these dynamic liquid droplets can undergo further phase transitions to form the less dynamic structures such as amyloid fibrils or inclusions that have been associated with various neurodegenerative diseases [6–17]. Consequently, LLPS lies at the heart of cellular physiology and pathology. Indeed, aggregation of FUS has been extensively observed in many neurodegenerative diseases, thus suggesting its general involvement in neurodegenerative diseases [3–17].

**Fig 1:**
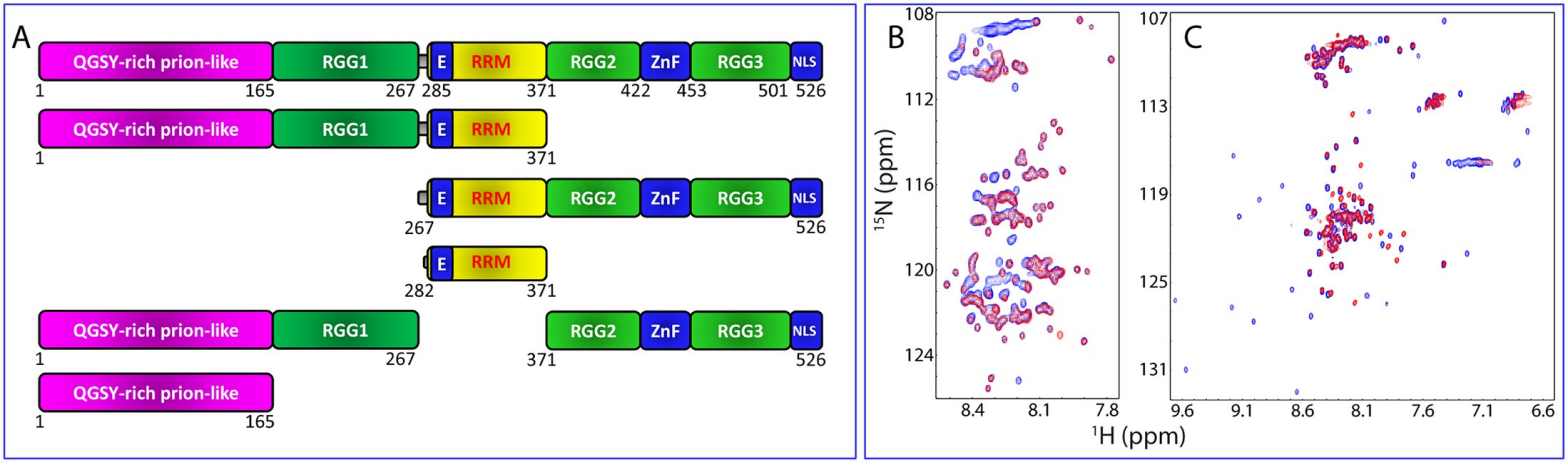
Domain organization of FUS and conformations of its NTD and CTD. (A) Domain organization of FUS and its six differentially dissected fragments used in the present study. 526-residue FUS is composed of an N-terminal LC region (1–267), including a QGSY-rich PLD over 1–165 and RGG1 over residues 166–267; RRM, 285–370; and CTD (371–526), which contains an RG/RGG-rich region over 371–422 (RGG2), a ZnF over 423–453, and another RG/RGG-rich region over 454–526 (RGG3) carrying an NLS. (B) Two-dimensional ^1^H-^15^N NMR HSQC spectra of the ^15^N-labeled FUS NTD (1–267) (blue) and PLD (1–165) (red) at 20 μM in 5 mM sodium phosphate buffer at pH 6.0. (C) HSQC spectra of the ^15^N-labeled FUS CTD (371–526) with the ZnF folded (blue) and unfolded (red) at 20 μM in 5 mM sodium phosphate buffer at pH 6.0. CTD, C-terminal domain; FUS, Fused in sarcoma; HSQC, Heteronuclear single quantum coherence spectroscopy; LC, low sequence complexity; NLS, nuclear localization signal; NTD, N-terminal domain; PLD, prion-like domain; QGSY, Gln-Gly-Ser-Tyr; RG/RGG-rich region, Arg-Gly/Arg-Gly-Gly–rich region; RRM, RNA-recognition motif; ZnF, zinc finger.

FUS is a 526-residue protein that is composed of three major domains (Fig 1A): the N-terminal domain (NTD) composed of the Gln-Gly-Ser-Tyr (QGSY)-rich prion-like domain (PLD) over 1–165 and an Arg-Gly (RG)/Arg-Gly-Gly (RGG)-rich region (RGG1) over 166–267, a middle RNA-recognition motif (RRM) over 282–371, and the C-terminal domain (CTD) over 371–526 containing RGG2, a zinc finger (ZnF), and RGG3 carrying a nuclear localization signal (NLS). FUS has two types of intrinsically disordered sequences with fundamentally distinct compositions of amino acids: namely, PLDs rich in polar residues including Tyr but completely absent of Arg/Lys and RGG regions abundant in Arg/Lys (S1A Fig). Interestingly, the FUS NTD represents an independent unit to fuse with CHOP with the DNA-binding domain to form an aberrant transcription factor TLS/FUS-CHOP [1,2], in which the FUS NTD is absolutely required for oncogenesis because the overexpression of only CHOP did not develop liposarcoma [18–20].

Recently, it was recognized that the formation and dissolution of LLPS in cells are under tight regulation by various cellular factors/events, and their misregulation will result in a large spectrum of human diseases [6–17,21–25]. In particular, LLPS and aggregation of RBDs have been revealed to be modulated by universal factors such as ATP [26,27] and nucleic acids [6–17,28–32]. ATP and nucleic acids share a common two-stage effect on LLPS of several RBPs, including FUS: enhancement of LLPS at low concentrations but dissolution at high concentrations. Now, nucleic acids have been established to play a general role in regulating LLPS of various proteins beyond RBPs [33–37].

Although physical models for phase transitions of biomacromolecules have been previously developed with the assumption that nonspecific interactions such as electrostatic interactions act as the key driving forces [38,39], many fundamental principles for LLPS in cells still remain unknown [14]. For example, it is still elusive how specific the driving forces are for LLPS of the protein components of the membraneless organelles. Why are only a small number of organelle types observed in eukaryotic cells, which contain N approximately 10^4^ types of proteins and nucleic acid molecules [14,39]? On the other hand, despite intense studies, so far, the high-resolution mechanisms have not been fully elucidated for LLPS. For example, the driving force for LLPS of FUS is still under debate; while previously, the N-terminal PLD (1–165) has been proposed to be the driver for LLPS of FUS mainly by the π–π interaction among the aromatic Tyr residues [7–11,29], a very recent study by systematic mutagenesis suggested that the regions critical for LLPS of FUS go beyond the PLD, and the major driving force was thus proposed to be the π-cation interaction [40]. In particular, so far, the mechanisms for the modulation of LLPS by ATP and nucleic acids remain completely undefined, and it even remains unknown whether ATP and nucleic acids modulate LLPS by nonspecific effect or specific interactions.

In the present study, by NMR spectroscopy guided by differential interference contrast (DIC) microscopy, we first assessed the driving forces for LLPS of FUS by systematically characterizing conformations, dynamics, and LLPS of full-length FUS and its six differentially dissected domains (Fig 1A) under a variety of conditions. Subsequently, we aimed to define the molecular mechanisms by which ATP and oligonucleic acids modulate LLPS of FUS by elucidating the molecular interactions of FUS and its domains with ATP, Adenosine monophosphate (AMP), and adenosine, as well as three oligonucleic acids, including one RNA and two single-stranded DNA (ssDNA) molecules with different secondary and tertiary structures. The results indicate that both types of intrinsically disordered domains, namely the PLD and RGG-rich regions, are needed for FUS to efficiently phase separate by establishing the π-cation interaction between aromatic residues within the PLD and Arg/Lys residues within RGG regions. Unexpectedly, we found that despite manifesting different NMR observations, both ATP and oligonucleic acids modulate LLPS of FUS and its dissected domains in the same manner, primarily by targeting the π-cation interactions through specific binding to Arg/Lys residues. Noticeably, oligonucleic acids have a much higher binding affinity and consequently are able to displace ATP from binding to FUS. The finding that the FUS CTD rich in Arg/Lys residues can be induced to phase separate followed by dissolution through the specific binding of the Arg/Lys residues with both ATP and oligonucleic acids implies that a myriad of proteins with the disordered regions rich in Arg/Lys residues might be also under modulation by ATP and nucleic acids in cells. Finally, our study also rationalizes the very recent discovery that Arg-rich C9ORF72 dipeptides become tightly associated with any nucleic acids to impair their functions to manifest cytotoxicity.

## Results

### LLPS of FUS and its differentially dissected domains

Previously, we have characterized the solution conformations of FUS and its differentially dissected domains fused with a 6×His-tag by circular dichroism (CD) and NMR [41]. However, our recent studies showed that although the short His-tag had no detectable effect on their solution conformations, it slightly weakened the capacity in LLPS, consistent with the recent report that the presence of the green fluorescent protein (GFP) tag significantly reduced the capacity in LLPS of FUS [40]. Therefore, in the present study, we prepared recombinant proteins without any tag residues and subsequently assessed LLPS of FUS and its six domains, namely FUS (1–165), FUS (1–267), FUS (1–371), FUS (282–371), FUS (267–526), and FUS (371–526) under a variety of conditions, which included different protein concentrations ranging from 1 to 500 μM; different pH values at 4.0, 6.0, and 6.8; and different buffers and salt concentrations up to 150 mM NaCl or KCl.

Full-length FUS was able to phase separate to form dynamic and spherical liquid droplets even at a protein saturation concentration of 2 μM, and at 20 μM, the maximal diameter of some droplets could reach approximately 3.7 μm (S1B Fig). By contrast, even after removing all tag residues, the PLD (1–165) still failed to phase separate at a protein concentration of 20 μM under various solution conditions. However, the PLD could phase separate to form small droplets with a maximal diameter of only approximately 1.0 μm at protein concentrations above 50 μM. Indeed, it has been previously shown that only at protein concentrations higher than 120 μM could the GFP-tagged PLD phase separate, while at lower protein concentrations, the addition of a molecular crowding agent, dextran, was needed to induce LLPS [29,40].

On the other hand, the entire NTD (1–267) with the additional RGG1 region (Fig 1A) could even phase separate at a protein saturation concentration of 1 μM without needing the addition of any crowding reagent. At 20 μM, it could form many droplets with a maximal diameter up to approximately 7.6 μm (S1C Fig). This suggests that the FUS NTD has much higher capacity in LLPS than the PLD. Interestingly, the PLD residues have very similar conformations in the isolated form and in the context of the NTD, as evidenced by the fact that almost all NMR HSQC peaks of PLD in the isolated form are superimposable to those of the corresponding residues in the context of the NTD at pH 6.0 (Fig 1B), as well as at pH 4.0 and 6.8 (S1D Fig). Furthermore, the isolated well-folded RRM showed no LLPS under a variety of conditions, and FUS (1–371) with both NTD and RRM could phase separate only at concentrations higher than 2 μM to form droplets with a maximal diameter of approximately 4.1 μm, implying that the additional presence of RRM would not enhance LLPS of NTD.

The FUS CTD over residue 371–526 is composed of RGG2, a ZnF, and RGG3 carrying an NLS (Fig 1A). As evidenced by its HSQC spectrum (Fig 1C), the ZnF domain is well folded, with many well-dispersed HSQC peaks. However, upon removing zinc cation by urea unfolding followed by Reverse-phase high-performance liquid chromatography (RP-HPLC) purification, the ZnF domain became unfolded, with all well-dispersed peaks disappearing (Fig 1C). Nevertheless, upon adding ZnCl into the unfolded sample even at a molar ratio of 1:2, the ZnF domain became spontaneously folded, with the HSQC spectrum indistinguishable from that of the native fold. We have thus characterized LLPS of the FUS CTD with both ZnF folded and unfolded under a variety of conditions, which included protein concentrations up to 500 μM, different pH values, and different salt types and concentrations with NaCl, KCL, and sodium phosphate concentration up to 150 mM. The results showed that the FUS CTD could not phase separate under these conditions. Furthermore, FUS (267–526) with the further inclusion of RRM also failed to phase separate under these conditions, indicating that the inclusion of RRM would not alter the intrinsic inability to phase separate for the FUS CTD.

### The enhancement of LLPS for NTD is not due to altered conformations and dynamics

**Fig 2:**
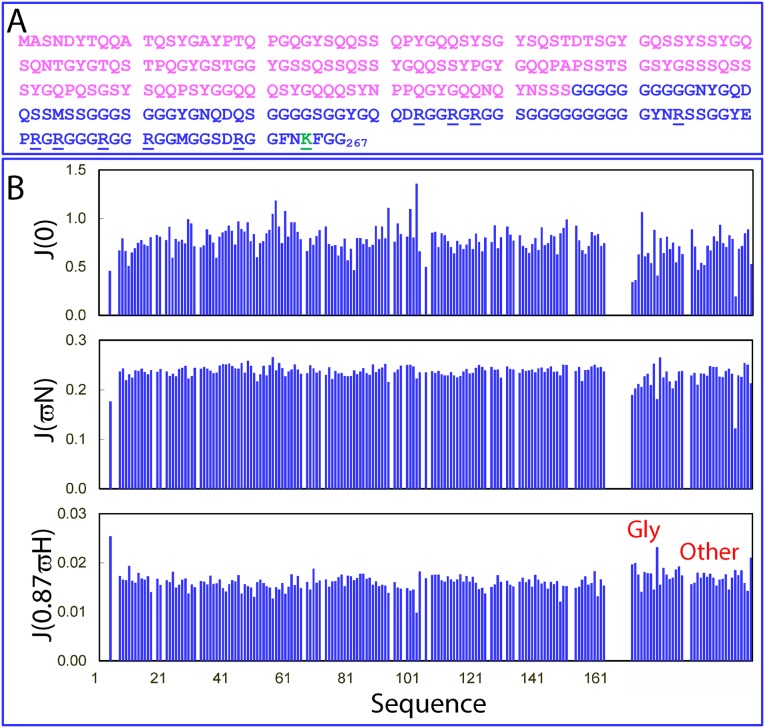
NMR backbone dynamics of the FUS NTD on a ps–ns timescale. (A) Amino-acid sequence of the FUS NTD (1–267) with the PLD (1–165) colored in purple and RGG1 (166–267) in blue. Arg residues are underlined, and one Lys residue is colored in green and underlined. (B) Spectral densities at J(0), J(ωN), and J(0.87ωH) of the ^15^N-labeled FUS NTD calculated from the ^15^N backbone relaxation data as measured at 800 MHz. FUS, Fused in sarcoma; NTD, N-terminal domain; PLD, prion-like domain; RG, Arg-Gly; RGG, Arg-Gly-Gly; RGG1, RG/RGG-rich region 1.

Our DIC studies showed that the FUS NTD is the minimal domain that can effectively phase separate. Very interestingly, it also represents an independent pathological domain, which could initiate liposarcoma development either by fusing or just coexpressing with CHOP [18–20]. Therefore, here we further characterized its conformation and dynamics by NMR spectroscopy. As shown in Fig 2A, the FUS NTD is composed of a PLD over residues 1–165 and RGG1 over residues 166–267. Previously, LLPS of the FUS PLD has been extensively characterized at pH 5.5 by NMR [29], but so far, no NMR study has been reported on the entire NTD. As such, the exact mechanism remains unknown as to why the inclusion of RGG1 significantly altered the capacity of LLPS because the addition of RGG1 might suddenly change the conformation and/or dynamics of the PLD, thus resulting in the enhanced capacity in LLPS.

Here, our results showed that the HSQC peaks of the PLD in the isolated form are almost completely superimposable to those of the corresponding residues in the NTD at pH 4.0, 6.0, and 6.8, respectively. Furthermore, the HSQC spectrum of our FUS PLD at pH 6.0 is also highly superimposable to that of the FUS PLD at pH 5.5 previously reported [29]. These results together indicate that the significantly enhanced capacity in LLPS of FUS NTD is not due to the conformational change of the PLD residues in the context of FUS NTD. So, we further characterized the dynamics of the entire NTD on different timescales by NMR.

Because of the extremely Gly-rich and highly degenerative and repetitive sequences of RGG1 (Fig 2A and S1A Fig), it is impossible to achieve NMR sequential assignment, as highlighted by a very recent report that only a partial assignment could be achieved for a 53-residue RGG-box of heterogeneous nuclear ribonucleoprotein A1 (hnRNPA1) [42]. As compared to the hnRNPA1 RGG-box, the FUS RGG1 is much longer (102 residues), higher in Gly-content (54.9%), and more degenerative and repetitive (Fig 2A). In particular, the FUS NTD has a high tendency to become further aggregated at a concentration higher than 50 μM. Therefore, it is impossible to assign FUS RGG1 residues with the currently available NMR methods.

We thus determined NMR backbone dynamics of the entire NTD on ps–ns and μs–ms timescales, which are very sensitive probes for disordered proteins but, on the other hand, whose interpretation needs no knowledge of types of amino acids [43–47]. Briefly, we acquired the ^15^N backbone relaxation data T1, T2, and [^1^H]-^15^N steady-state heteronuclear Nuclear Overhauser Effect (hNOE) (S2 Fig), as well as Carr–Purcell–Meiboom–Gill (CPMG) relaxation dispersion data for the NTD at 50 μM in 5 mM sodium phosphate buffer (pH 6.0). In the analysis, we used the previous assignment for the PLD [29] but divided the unassigned residues over 166–267 into two categories: Gly residues with their HSQC peaks well-separated and distinguishable from the rest of peaks and other (non-Gly) residues. As indicated by the relaxation data, particularly hNOE (S2 Fig), the PLD residues are also highly disordered in the context of the entire NTD, very similar to what was previously reported for the isolated FUS PLD [29]. Moreover, the NMR relaxation data also provided critical insights into the dynamics of the extra Gly-rich region 166–271: both Gly and other non-Gly residues are also disordered, highly similar to the PLD residues.

We subsequently calculated reduced spectral densities at three frequencies—0, ωN, and 0.87ωH (Fig 2B)—from the ^15^N backbone relaxation data (S2 Fig), which reflect relaxation contributions from motions/exchanges on different timescales [43,44,47]. As shown in Fig 2B, the low J(0) and high J(0.87ωH) values over almost the whole sequence 1–267 indicate that in the entire NTD, both PLD and RGG1 residues are highly disordered, with a similar backbone flexibility on the ps–ns timescale. Furthermore, analysis of the CPMG data also showed that all resolved peaks have no significant dispersion (>2 Hz) over the range from 40 Hz to 980 Hz [43,45,46], suggesting that for the entire NTD, which has much higher capacity in LLPS, the monomeric state and phase-separated state are also undergoing conformational exchanges faster than a millisecond, similar to what was previously reported for the isolated PLD in the presence of the crowding reagent [29]. Moreover, we also conducted NMR self-diffusion measurements on the FUS NTD [48–50], and a diffusion coefficient of approximately 0.60 ± 0.01 × 10^−10^ m^2^/s was obtained, which is very similar to that of transferrin with a molecular mass of approximately 80 kDa [49]. Therefore, NMR results on conformation, dynamics, and self-diffusion together suggest that the FUS NTD is intrinsically disordered without any tight packing [43–50].

Our NMR data thus indicate that the significant enhancement of the capacity in LLPS of the entire NTD is not due to the significant changes of the conformations and dynamics of PLD in the context of the entire NTD. Therefore, the enhanced capacity should mainly result from the additional interaction established upon introducing RRG1. As shown in S1A Fig, the FUS PLD contains a large number of polar residues, including Gln, Ser, and Thr as well as 24 aromatic residues Tyr (14.5%), but completely lacks Arg and Lys residues. For RGG1, all types of amino acids can be found in PLD except for 9 Arg (8.8%) and 1 Lys (1%) residues. Because Tyr residues within the FUS PLD have been systematically demonstrated to be critical for LLPS of FUS [40], one possible explanation for the enhanced capacity in LLPS observed on the FUS NTD is that the additional inclusion of RGG1 leads to the establishment of new π-cation interactions between aromatic Tyr residues within the PLD and Arg/Lys residues within RGG1 [40,51,52]. Therefore, the π-cation interactions newly established between Tyr within the PLD and Arg/Lys within RGG1 could represent the major driving force for LLPS of the FUS NTD, completely consistent with the systematic mutagenesis results [40].

### Zinc coordination drives the folding of the ZnF domain within the FUS CTD

So far, the residue-specific conformation has never been experimentally characterized for the entire FUS CTD (Fig 1A and S3A Fig). Therefore, here we attempted to determine the residue-specific solution conformation of the FUS CTD by NMR spectroscopy. Because the FUS CTD is highly soluble with a concentration even up to 500 μM, we have successfully acquired and analyzed a large set of three-dimensional NMR spectra, including cross-bond triple-resonance HN(CO)CACB, CCC(CO)NH, and HNN as well as cross-space HSQC-Total Correlation Spectroscopy (TOCSY) and HSQC-Nuclear Overhauser Effect Spectroscopy (NOESY) experiments. In particular, the FUS CTD has a sequence much less degenerative than RGG1, as well as containing a folded ZnF with well-dispersed HSQC peaks. Consequently, we have achieved the assignment for detected HSQC peaks (S1 Table). The assignment revealed that indeed, all well-dispersed HSQC peaks are from the residues of the folded ZnF over 421–454, while RGG2, RGG3, and NLS residues are highly disordered, thus having narrowly dispersed HSQC peaks (S3B Fig).

The chemical shift index (CSI) of Cα, Cβ, and Hα protons—the difference between the experimental and random coil chemical shifts—is a sensitive indicator of secondary structure population for both folded and unfolded proteins [43,47,50,53]. As shown in S3C and S3D Fig, except for the ZnF residues, all FUS CTD residues have very small absolute values of (ΔCα–ΔCβ) and Hα CSI, which strongly suggests that the CTD regions other than ZnF are highly disordered without any stable secondary structures. To gain quantitative insights into the populations of different secondary structures, we further analyzed NH, N, Hα, Cα, and Cβ chemical shifts of the FUS CTD by Secondary Structure Propensity (SSP) program [53]. As seen in S3E Fig, all residues other than the ZnF residues have absolute values smaller than 0.4, indicating that they indeed have no stable secondary structures.

**Fig 3:**
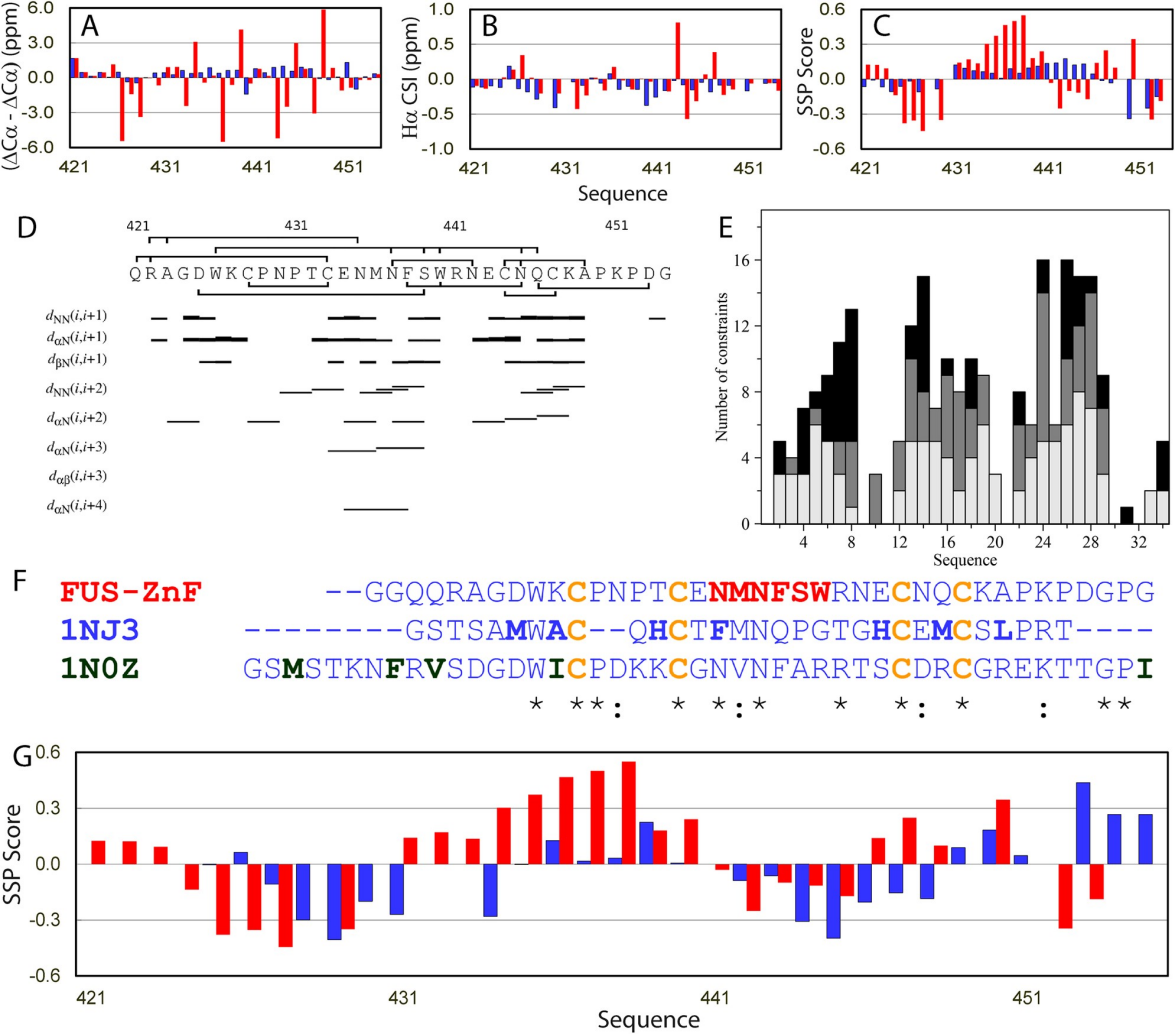
Residue-specific conformations of the FUS CTD with ZnF folded and unfolded. Residue-specific values of the unfolded (blue) and folded (red) ZnF residues over 421–454 for (ΔCα–ΔCβ) (A), (ΔHα) (B), and SSP (C). (D) Sequence of the folded ZnF over residue Gln321–Gly354 with assigned NOE displayed. (E) Distribution of NOEs over the sequence of the folded FUS ZnF (white bars: sequential NOEs, gray bars: medium-range NOEs, and black bars: long-range NOEs). (F) Sequence alignment between the FUS ZnF and two other ZnF domains. (G) SSP values of the folded FUS ZnF (red) and Npl4 ZnF (blue). CTD, C-terminal domain; FUS, Fused in sarcoma; NOE, Nuclear Overhauser Effect; SSP, Secondary Structure Propensity; ZnF, zinc finger.

A close examination of the CSI (Fig 3A and 3B) and SSP values (Fig 3C) of the ZnF residues 421–454 indicates that residues 425–429 have SSP values <−0.3, implying that they might adopt extended conformations; while residues 434–438 have SSP values >0.3, implying that they may assume helical conformations. Further analysis of the HSQC-NOESY spectrum revealed the existence of a network of NOEs that includes 50 sequential, 29 medium-range, and 26 long-range NOEs over the ZnF residues (Fig 3D and 3E). Because the FUS ZnF belongs to the C4 subfamily of the ZnF superfamily [54], we thus downloaded and analyzed all crystal and NMR structures of the C4 ZnF proteins and found that two ZnF domains have high sequence homology with the FUS ZnF [Fig 3F]: one is the conserved ZnF of Npl4 protein with Protein Data Bank (PDB) ID of 1NJ3 [55], while another is the ZnF from human splicing factor ZNF265 with PDB ID of 1N0Z [56]. However, 96 long-range NOEs have been identified for 1NJ3 [55] and 114 for 1N0Z [56], which are much more than the number identified for the FUS ZnF. Because only the NMR structure of Npl4 ZnF was determined by heteronuclear NMR spectroscopy with chemical shifts deposited, we downloaded its chemical shifts and analyzed them by SSP program. The results showed that despite having high sequence homology, the FUS and Npl4 ZnF have significantly different secondary structure propensity: the Npl4 ZnF is characteristic of two extended fragments, while FUS ZnF appears to have a unique well-formed helical fragment (Fig 3G).

A detailed examination of their sequences revealed that the FUS ZnF contains no large hydrophobic residues Val, Ile, or Leu, while other two have both large hydrophobic residues and more aromatic residues. This implies that the folding of the FUS ZnF might be predominantly driven by Zn-coordination. To confirm this, we denatured the FUS CTD by 8 M urea followed by purification with reverse-phase HPLC to remove zinc ion. The zinc-depleted FUS CTD had no well-dispersed HSQC peaks (Fig 1C). On the other hand, the FUS CTDs with the folded and unfolded ZnF have completely superimposable HSQC peaks for all residues except for residues 421–454. We also collected the three-dimensional NMR spectra and successfully assigned the FUS CTD with the unfolded ZnF (S1 Table). Indeed, the zinc-depleted FUS ZnF residues all have the absolute values of SSP <0.2 (Fig 3C), indicating that the FUS ZnF is incapable of folding by itself in the absence of Zn-coordination, most likely because of the lack of large hydrophobic residues. Consequently, even the folded FUS ZnF coordinated with Zn has no very tight tertiary packing and is thus short on long-range NOEs. Furthermore, we also found no slowly exchanged backbone amide protons for FUS CTD even with the folded ZnF. This also explains why the previous NMR study of the isolated FUS ZnF domain failed to determine its solution structure but only generated a homology model with the Npl4 ZnF [57]. Interestingly, this is reminiscent of our previous observation that even when the tight side-chain packing of a 37-residue small protein was largely disrupted by acid unfolding, many long-range NOEs disappeared. On the other hand, it is still able to maintain the native-like tertiary topology mainly by the strong covalent constraints provided by two disulfide bridges [58].

### RNA and ssDNA have the same two-stage effects on LLPS of FUS

Recently we found that in addition to dissolving LLPS of FUS as a hydrotropic molecule at high concentrations [26], ATP could also enhance LLPS of FUS at low concentrations [27]. As a consequence, both ATP and RNA have been shown to exhibit the same two-stage effect on LLPS of FUS: enhancement at low ATP concentrations but dissolution at high concentrations. On the other hand, although ssDNA was found to have the same two-stage effect on the TAR DNA-binding protein 43 (TDP-43) domains [31], it remains unknown whether ssDNA also has the same effect on LLPS of FUS.

**Fig 4:**
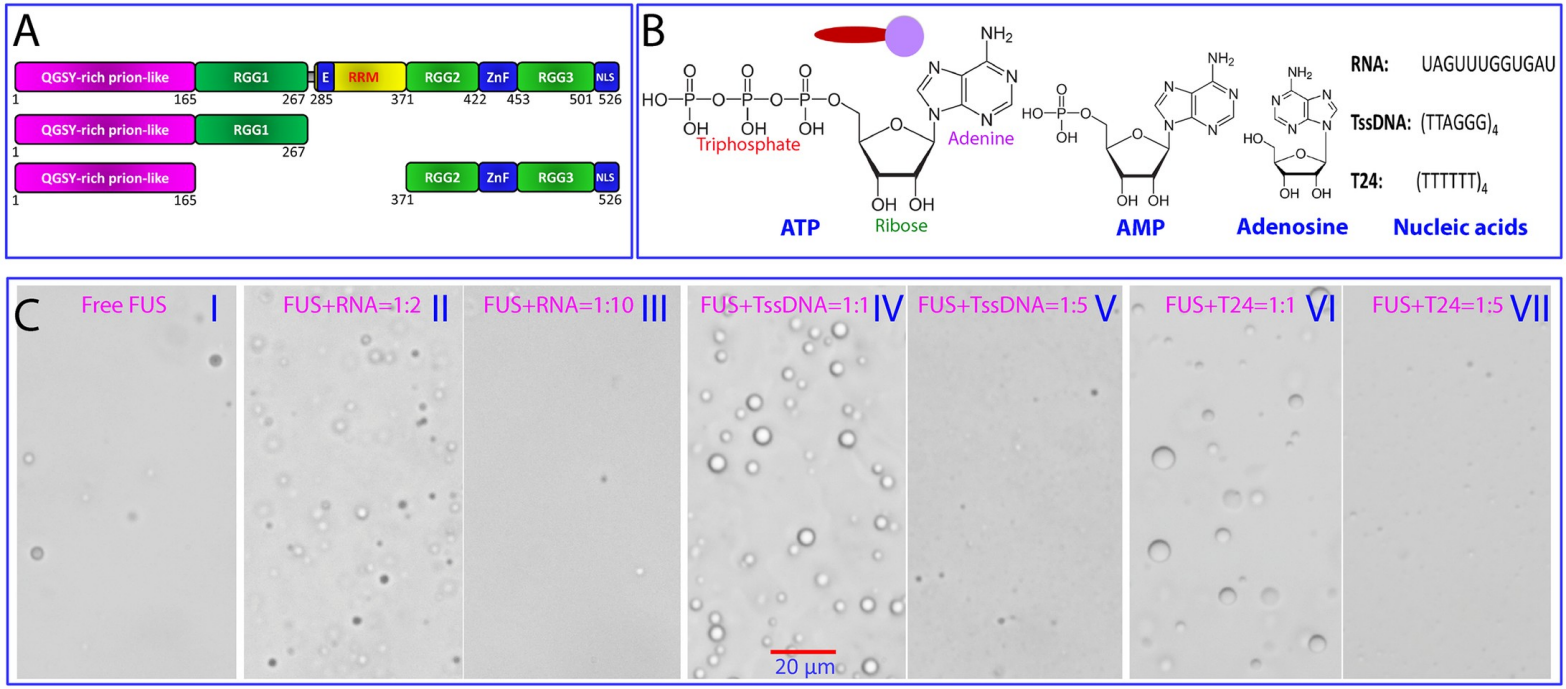
LLPS of FUS as modulated by ATP and nucleic acids. (A) FUS and its domains used in the present study for characterizing the modulation of their LLPS by ATP, AMP, and adenosine, as well as one RNA and two ssDNA molecules (B). (C) DIC microscopy images of liquid droplets formed by FUS in the presence of RNA and two ssDNA at different molar ratios. The videos for outputting these images are provided in Supporting Information. AMP, Adenosine monophosphate; DIC, differential interference contrast; FUS, Fused in sarcoma; LLPS, liquid–liquid phase separation; NLS, nuclear localization signal; QGSY, Gln-Gly-Ser-Tyr; RGG-rich region, Arg-Gly-Gly–rich region; RRM, RNA-recognition motif; ssDNA, single-stranded DNA; TssDNA, telomeric ssDNA; ZnF, zinc finger.

Here, we further conducted a systematic NMR and DIC characterization of LLPS of FUS and its three domains (Fig 4A), namely the PLD (1–165), the entire NTD (1–267), and the entire CTD (371–526), with the ZnF either folded (F-CTD) or unfolded (U-CTD) in the presence of ATP, AMP, and adenosine, as well as three oligonucleic acids, including one RNA and two ssDNA molecules (Fig 4B). This study was conducted under an optimized condition, namely at 20 μM protein concentration in 5 mM sodium phosphate (pH 6.0) with 1 mM MgCl_2_, which was previously used for characterizing the mediation of FUS LLPS by ATP [27]. In order to assess the effects of different nucleic acids, here we selected three oligonucleic acid molecules (Fig 4B), one RNA with a sequence of UAGUUUGGUGAU and the telomeric ssDNA (TssDNA) with a sequence of (TTAGGG)_4_, because both of them have been previously shown to have the functionally relevant binding to the FUS domains by various methods including NMR [57,59]. However, because TssDNA is able to form a G-quadruplex tertiary structure [57,59,60], we thus further included ssDNA T24 as a control that is supposed not to form any secondary and tertiary structures.

We first assessed the conformations of two ssDNA molecules in different buffer conditions by far-UV CD spectroscopy (S4 Fig). Indeed, T24 showed no formation of any secondary and tertiary structures even with an NaCl concentration up to 150 mM (S4A Fig), while TssDNA adopted the typical G-quadruplex structure in the 5 mM sodium phosphate buffer (S4B Fig) as previously characterized [60]. Subsequently, we characterized LLPS of FUS in the presence of RNA or ssDNA at different molar ratios, including 1:0.1, 1:0.5, 1:1, 1:2, 1:5, 1:10, :1:15, 1:20, 1:25, 1:30, 1:35, 1:40, 1:45, and 1:50 (FUS:RNA/ssDNA) as monitored by DIC microscopy. In the absence of nucleic acids, FUS itself was able to undergo LLPS to form only a small number of droplets with a maximal diameter of approximately 3.7 μm (I of Fig 4C and S1 Video). By contrast, addition of RNA at a ratio of 1:2 significantly enhanced LLPS by mainly increasing the numbers of droplets with a maximal diameter of approximately 4.0 μm (II of Fig 4C and S2 Video). Further addition to 1:10 led to significant dissolution of liquid droplets by reducing both numbers and sizes of droplets with a maximal diameter of only approximately 2.5 μm (III of Fig 4C and S3 Video). When the ratio reached 1:15, the droplets completely disappeared. Interestingly, in a recent report [32], for GFP-tagged FUS at 5 μM, a concentration of 100 ng/μl of total RNA was needed to enhance LLPS, while a concentration of 300 ng/μl significantly induced dissolution. In our current study, 38 ng/μl of 12-mer RNA was able to significantly enhance LLPS of tag-free FUS, while 190 ng/μl of the RNA was sufficient to induce significant dissolution. This difference is likely due to the fact that in total RNA, some RNA molecules might not bind or only weakly bind to FUS, and consequently the averaged capacity of total RNA is slightly weaker than that of the pure RNA we used here in both induction and dissolution of FUS LLPS.

We next assessed the effect of two ssDNA molecules under exactly the same solution conditions. For TssDNA, at a molar ratio of 1:1 (FUS:TssDNA), LLPS of FUS appeared to be significantly enhanced by forming a large number of droplets with a maximal diameter up to 5.4 μm (IV of Fig 4C and S4 Video). Further addition to 1:5 led to significant dissolution of droplets with the maximal diameter reduced to only approximately 1.6 μm (V of Fig 4C and S5 Video), and at 1:10, all droplets were completely dissolved. Interestingly, T24 showed the same two-stage effect on LLPS of FUS as TssDNA. At a molar ratio of 1:1, LLPS of FUS was significantly enhanced by forming a large number of droplets with a maximal diameter up to approximately 6.2 μm (VI of Fig 4C and S6 Video). Similarly, addition to 1:5 led to significant dissolution of droplets with the maximal diameter reduced to approximately 1.2 μm (VII of Fig 4C and S7 Video), and at 1:10, all droplets were also dissolved. This implies that the secondary and tertiary structures of ssDNA in the free state are not essential for achieving the two-stage effect on LLPS of FUS.

Here, we showed that one RNA and two ssDNA molecules with distinctive structures are all able to modulate LLPS of FUS in the same two-stage manner. The effect appears to be dependent on the binding affinities of nucleic acids with FUS. Indeed, previously, FUS has been shown to bind various nucleic acids, including RNA and ssDNA, with highly degenerative specificity to types and sequences of nucleic acids [57,59,61,62]. Interestingly, two 24-mer ssDNA molecules have higher capacity than the 12-mer RNA in mediating LLPS of FUS. This appears to be correlated to their length-dependent binding affinity to FUS domains. For example, for the FUS RRM domain, a dissociation constant (Kd) of 422 μM was obtained for this RNA and 214 μM for TssDNA, respectively [59]. This suggests that the capacity of nucleic acids in modulating LLPS of FUS is mainly correlated to their binding affinity. Therefore, for NMR experiments that need a large quantity of nucleic acids as well as long experimental times, we only used two 24-mer ssDNA molecules because they have a stronger capacity for mediating LLPS and higher chemical stability, as well as being much less costly. Previously, we have observed that RNA molecules became degraded during NMR experiments, most likely because of the contamination by the extremely stable ribonuclease.

### ATP and oligonucleic acids monotonically dissolve LLPS of NTD by specifically binding

Recently, we found that ATP could monotonically dissolve LLPS of the FUS NTD, as characterized by the intensity reduction of all HSQC peaks [27]. Further analysis here showed that the intensity of HSQC peaks uniformly reduced over the whole sequence upon gradual addition of ATP. To understand the requirement of different groups of ATP molecules in mediating LLPS, here we further titrated the FUS NTD with AMP and adenosine under the same condition. The results showed that AMP could also induce the uniform reduction of the HSQC peak intensity but was much less effective than ATP: even at 10 mM, only a slight reduction was observed (S5A Fig), and only at 50 mM could the droplets formed by NTD be dissolved, characterized by the disappearance of most HSQC peaks. On the other hand, adenosine triggered no significant change of the HSQC peak intensity even at 10 mM (S5B Fig) as well as no detectable dissolution of the droplets at 10 mM. Further attempts to increase the adenosine concentration failed because of its low solubility in buffers. Nevertheless, the results clearly indicate that the capacity of ATP to dissolve LLPS of NTD is highly dependent on the presence of the triphosphate chain, and its removal significantly abolished the capacity.

Subsequently, we assessed the effects of three oligonucleic acids on LLPS of the entire NTD at different molar ratios, including 1:0.1, 1:0.5, 1:1, 1:2, 1:5, 1:10, :1:15, 1:20, 1:25, 1:30, 1:35, 1:40, 1:45, and 1:50, by DIC imaging (S6A Fig). Interestingly, like ATP, RNA and two ssDNA molecules also induced the same monotonic dissolution of LLPS of NTD. Out of three oligonucleic acids, the highest concentrations were needed for RNA to achieve the similar dissolution of LLPS induced by two ssDNA molecules. Briefly, a ratio of 1:2 was required for RNA (S6B Fig), but only a ratio of 1:1 was sufficient for two ssDNA molecules to significantly disrupt LLPS to reduce the sizes of droplets, with the maximal diameter reduced from approximately 7.6 to < 3.0 μm (S6C and S6D Fig). Furthermore, a ratio of 1:5 was required for RNA (0.1 mM), but only a ratio of 1:2 was sufficient for two ssDNA molecules (0.04 mM) to completely dissolve LLPS of the NTD.

**Fig 5:**
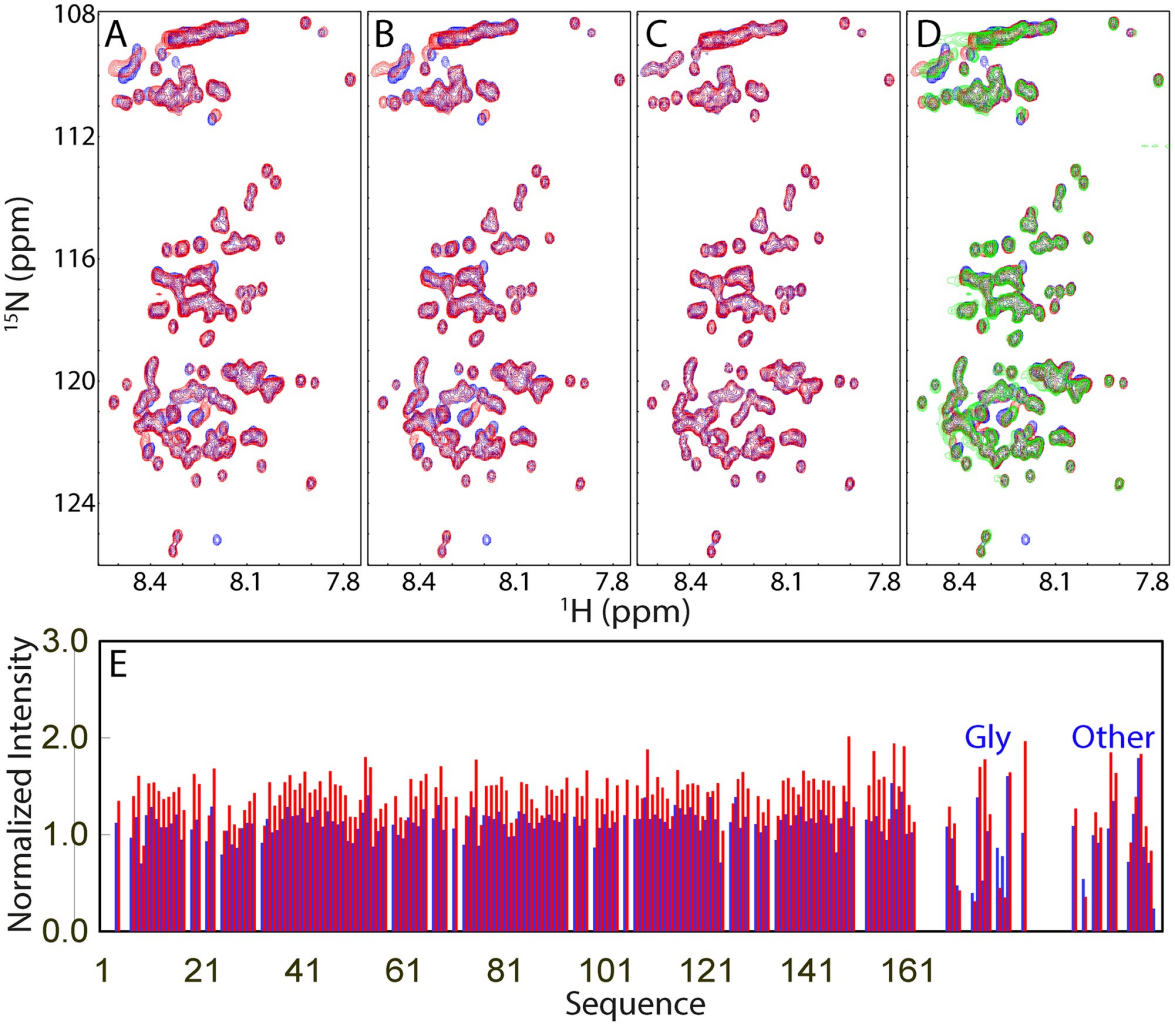
NMR view of the dissolution of LLPS of the FUS NTD induced by ssDNA. HSQC spectra of the ^15^N-labeled FUS NTD in the absence (blue) and in the presence of TssDNA (red) at a ratio of 1:1 (A) and at 1:2 (B). (C) HSQC spectra of the ^15^N-labeled FUS NTD in the presence of TssDNA at a ratio of 1:2 (blue) and at 1:5 (red). (D) HSQC spectra of the ^15^N-labeled FUS NTD in the absence (blue) and in the presence of TssDNA at a ratio of 1:5 (red) and of T24 at 1:5 (green). (E) Normalized HSQC peak intensity of the ^15^N-labeled FUS NTD in the presence of TssDNA at molar ratio of 1:0.1 (blue) and 1:2 (red) as divided by that of the FUS NTD in the free state. FUS, Fused in sarcoma; HSQC, Heteronuclear single quantum coherence spectroscopy; LLPS, liquid–liquid phase separation; NTD, N-terminal domain; ssDNA, single-stranded DNA; TssDNA, telomeric ssDNA.

To gain a residue-specific view of the interaction between the FUS NTD and ssDNA in mediating LLPS, we characterized dissolution of the liquid droplets of the FUS NTD induced by two ssDNA molecules with NMR HSQC titrations under exactly the same solution condition and at the same molar ratios as used for the above DIC imaging. Interestingly, at a molar ratio of 1:1, only a small set of HSQC peaks either became shifted or disappeared (Fig 5A). Addition of TssDNA to 1:2 only led to further slight shifting of several HSQC peaks (Fig 5B). No further shift or disappearance of HSQC peaks was detected at 1:5 (Fig 5C) and even up to 1:50. Addition of T24 also triggered the shift and disappearance of the same set of HSQC peaks (Fig 5D), implying that both TssDNA and T24 bind to the same set of residues. Superimposition of HSQC spectra of the isolated PLD and entire NTD revealed that the residues with shifted or disappeared HSQC peaks upon adding TssDNA and T24 are all located within RGG1, implying that the PLD has no detectable binding to two ssDNA molecules.

We then analyzed the intensity change of HSQC peaks of the NTD (Fig 5E) in the presence of TssDNA. As shown in Fig 5E, for the PLD residues, addition of TssDNA at a ratio of 1:0.1 led to a slight increase of the intensity with an average of 1.2 times higher than that without ssDNA. By contrast, for the RGG1 residues, addition of ssDNA triggered three types of significant changes: some residues have their HSQC peak intensity reduced or even disappear, while several other have their HSQC peak intensity increased. At a ratio of 1:2, the intensity of HSQC peaks of PLD further increased with an average of 1.4 times higher than that in the free state, while the RGG1 residues still showed differential changes (Fig 5E). Further addition of TssDNA to high ratios led to no significant difference from that at 1:2.

The present NMR results of the NTD titrated with two ssDNA molecules are unexpected in consideration of the fact that ATP is one building block of nucleic acids, as well as both ATP and ssDNA inducing the same monotonic dissolution of LLPS of NTD. So why are the patterns of the intensity changes of the NTD HSQC peaks induced by ATP and oligonucleic acids so different? While ssDNA induced the differential intensity changes of HSQC peaks for PLD and RGG1, ATP triggered the uniform intensity reduction of HSQC peaks of all NTD residues. To understand the underlying mechanisms for this observation, we titrated the PLD at 20 μM in the same buffer with ATP and two ssDNA molecules as monitored by both DIC imaging and NMR. The results showed that at 20 μM, no LLPS was induced for the PLD by adding ATP up to 10 mM as well as two ssDNA molecules, even at a ratio up to 1:50 (1 mM). Consistent with DIC results, for ATP, only very minor shifts of several HSQC peaks were observed, but no significant change of the peak intensity was found for PLD even in the presence of ATP at 10 mM (S5C Fig). Furthermore, for ssDNA, no significant shift or intensity change of HSQC peaks was detected upon adding two ssDNA molecules as exemplified by TssDNA at a ratio of 1:50 (1 mM) (S5D Fig). The results imply that the π–π interaction between the aromatic rings of Tyr within the PLD and the base aromatic rings of ATP/oligonucleic acids is also very weak and thus insufficient to induce LLPS of the PLD.

**Fig 6:**
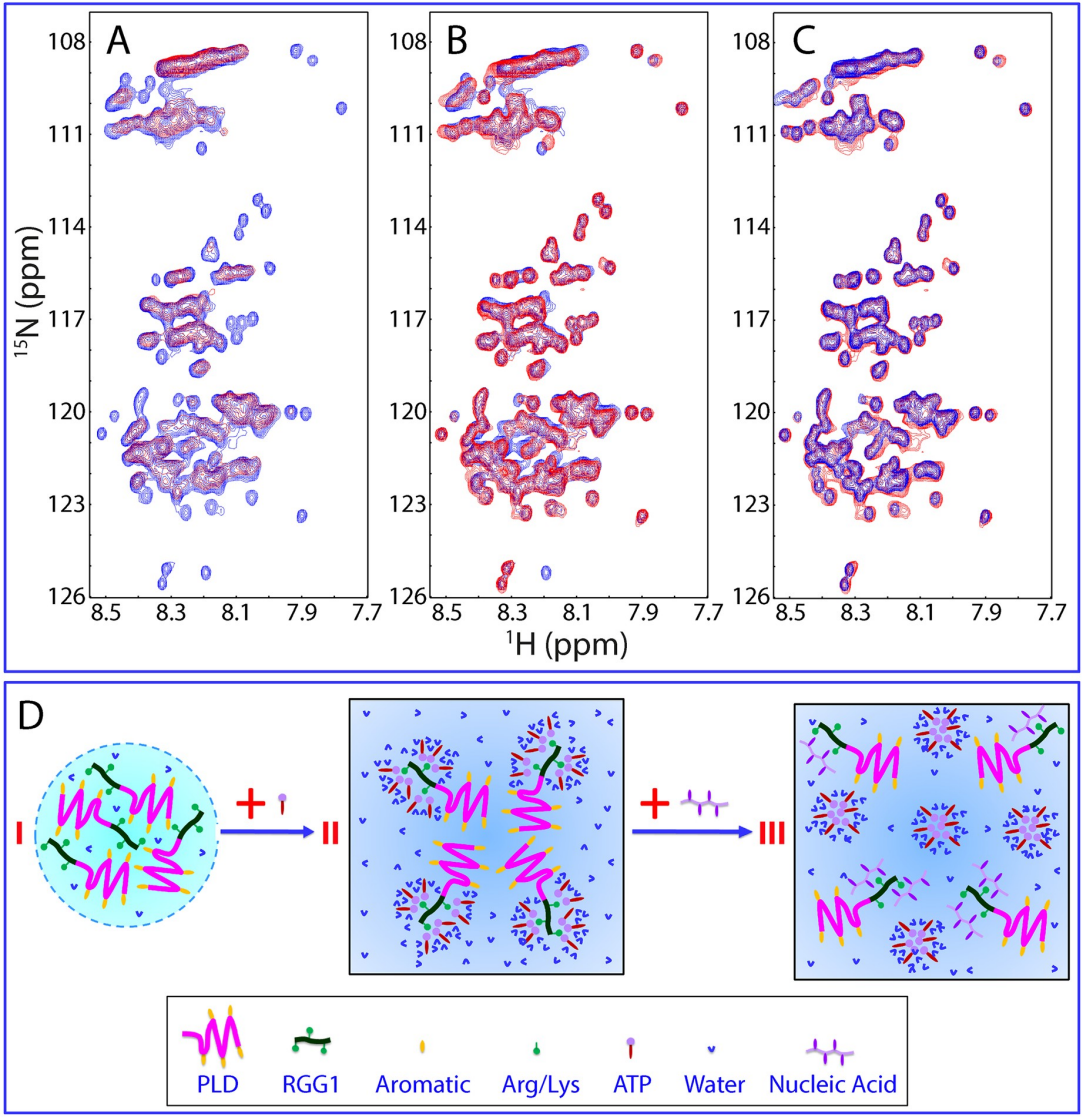
ssDNA can displace ATP from binding the FUS NTD. (A) HSQC spectra of the ^15^N-labeled FUS NTD in the absence (blue) and in the presence of ATP at 3 mM (red). (B) HSQC spectra of the ^15^N-labeled FUS NTD in the absence (blue), and in the presence of ATP at 3 mM with an extra addition of TssDNA at 60 μM (red). (C) HSQC spectra of the ^15^N-labeled FUS NTD in the presence of TssDNA at a ratio of 1:5 (blue) and in the presence of both ATP at 3 mM and TssDNA at 60 μM (red). (D) A speculative model to rationalize the specific binding of ATP to Arg/Lys residues within RGG1 of the FUS NTD, which, however, can be displaced by ssDNA. FUS, Fused in sarcoma; HSQC, Heteronuclear single quantum coherence spectroscopy; NTD, N-terminal domain; PLD, prion-like domain; RGG1, Arg-Gly-Gly–rich domain 1; ssDNA, single-stranded DNA; TssDNA, telomeric ssDNA.

The results also imply that ATP and two ssDNA molecules dissolved LLPS of the FUS NTD by binding to the same or at least highly overlapped set of the residues over RRG1. To test this hypothesis, we added ATP into the FUS NTD sample to reach a final concentration of 3 mM to mimic the ATP concentration in neuron cells of approximately 2.7 mM [63], and consequently, the HSQC peak intensity was significantly reduced (Fig 6A). Subsequently, we gradually added TssDNA or T24 into this sample at ratios of 1:0.1, 1:0.5, 1:1, 1:2.5, 1:5, 1:10, 1:25, and 1:50 (NTD:TssDNA/T24). Upon adding TssDNA at a ratio of 1:2.5, corresponding to 0.05 mM, many HSQC peaks reappeared (Fig 6B), and further addition up to 1:50 showed no significant changes. Most unexpectedly, the HSQC spectrum of the NTD in the presence of both ATP at 3 mM and TssDNA at 0.05 mM is highly superimposable to that of the NTD saturated by binding to ssDNA alone (Fig 6C). This observation thus suggests that 1) indeed, the RGG1 residues for binding ATP and ssDNA are the same or at least highly overlapped; and 2) the binding affinity of ssDNA to these residues is much higher than that of ATP, and consequently, ssDNA at 0.05 mM was sufficient to displace ATP at 3 mM from binding to the FUS NTD.

So, an interesting question arises: if ATP and two ssDNA both bind to the same set of residues within RRG1 to dissolve LLPS of the FUS NTD, why does the binding of ATP induce uniform intensity reduction of HSQC peaks of all NTD residues? Here, we propose that this unique NMR observation for ATP is most likely due to the hydrotropic nature of ATP as previously reported [26,64–68]. ATP has been previously shown to be able to self-assemble into oligomers, which is highly dependent on the presence of the triphosphate group and can strongly interact with water molecules [26,64–68]. Therefore, the Arg/Lys residues within RGG1 critical for interacting with aromatic residues within PLD to phase separate (I of Fig 6D) can also interact with ATP by establishing the π-cation interaction between the base aromatic ring of ATP and Arg/Lys residues within RGG1. When the ATP concentration is sufficiently high, the adenine aromatic ring of ATP will be clustered around Arg/Lys residues within RGG1. However, different from oligonucleic acids, the triphosphate group of ATP will strongly interact with water molecules. Consequently, the NTD molecules bound with ATP will further self-assemble into large and dynamic oligomers in which the aromatic/hydrophobic PLD residues are also involved, although they have no direct binding with ATP (II of Fig 6D). Because of the size and dynamics of the ATP-NTD oligomers, most NMR signals become too broad to be detected. However, upon adding ssDNA molecules that have a much higher affinity to Arg/Lys residues, the ATP molecules will be displaced from being clustered around Arg/Lys residues, and the ATP-NTD oligomers will be disassembled (III of Fig 6D). Consequently, the NTD molecules become bound with ssDNA, and most NMR HSQC peaks will become detectable.

### ATP and oligonucleic acids induce and then dissolve LLPS of the FUS CTD by specific binding

Both the FUS F-CTD and U-CTD lack the intrinsic capacity to phase separate. However, we recently found that ATP could induce the FUS F-CTD to phase separate at 1–2 mM, followed by subsequent dissolution at high ATP concentrations >4 mM in vitro [27]. Detailed analysis showed that ATP also induced the uniform intensity reduction of HSQC peaks of FUS F-CTD residues, and consequently, the binding residues remained unknown. In an attempt to identify the binding residues, here we further titrated the FUS F-CTD with AMP and adenosine but found that they were unable to induce LLPS even at a concentration of AMP up to 50 mM. NMR HSQC characterization showed that both AMP (S7A Fig) and adenosine (S7B Fig) only have very weak interactions with the F-CTD, as indicated by slight shifts of several HSQC peaks.

**Fig 7:**
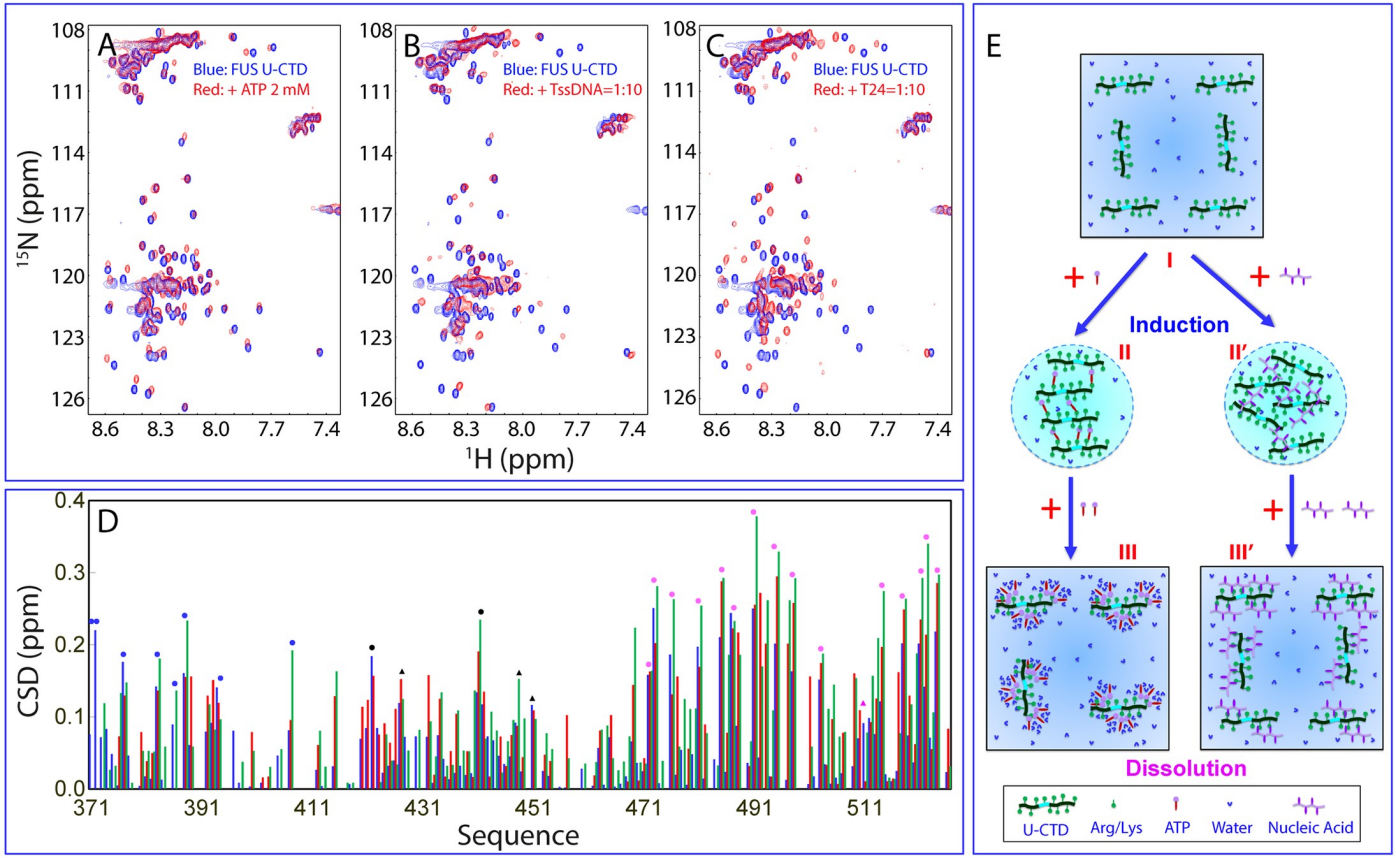
NMR view of the induction and dissolution of LLPS of the FUS U-CTD. (A) HSQC spectra of the ^15^N-labeled FUS U-CTD in the absence (blue) and in the presence of ATP at 2 mM (red). (B) HSQC spectra of the ^15^N-labeled FUS U-CTD in the absence (blue) and in the presence of TssDNA at a ratio of 1:10 (red). (C) HSQC spectra of the ^15^N-labeled FUS U-CTD in the absence (blue) and in the presence of T24 at a ratio of 1:10 (red). (D) CSD of the FUS U-CTD in the presence of ATP at 2 mM (blue) and in the presence of TssDNA (red) and T24 (green) at a ratio of 1:10. Filled circles are used for indicating the locations of Arg residues, while triangles indicate Lys. Blue is for those within RGG2, black for those in the unfolded ZnF, and purple for those within RGG3. (E) A speculative model to rationalize the specific binding of ATP and ssDNA to Arg/Lys residues within the FUS U-CTD to induce LLPS at low concentrations but to dissolve at high concentrations. CSD, chemical shift difference; CTD, C-terminal domain; FUS, Fused in sarcoma; HSQC, Heteronuclear single quantum coherence spectroscopy; LLPS, liquid–liquid phase separation; RGG, Arg-Gly/Arg-Gly-Gly–rich region; ssDNA, single-stranded DNA; TssDNA, telomeric ssDNA; U-CTD, ZnF unfolded CTD; ZnF, zinc finger.

Subsequently, here we titrated the U-CTD with ATP and found that ATP was also able to induce and then dissolve LLPS with the same pattern as we previously observed on the F-CTD [27]. Strikingly, however, we were able to detect the shifts of HSQC peaks of the U-CTD residues upon gradual addition of ATP. Interestingly, at 2 mM, the shifts of HSQC peaks were mostly saturated (Fig 7A), and at higher ATP concentrations, up to 10 mM, only reduction of peak intensity was observed. This thus allowed the calculation of the chemical shift difference (CSD) of the FUS U-CTD in the free state without LLPS and in the presence of ATP at 2 mM with significant LLPS (Fig 7D).

On the other hand, we also characterized the effects of one RNA and two ssDNA molecules on LLPS of F-CTD at different molar ratios, including 1:0.1, 1:0.5, 1:1, 1:2, 1:5, 1:10, :1:15, 1:20, 1:25, 1:30, 1:35, 1:40, 1:45, and 1:50. Upon adding RNA at a molar ratio of 1:2, many small droplets formed with a maximal diameter of approximately 1.1 μm (I of S8A Fig and S8 Video). However, at 1:5, droplets were significantly dissolved, with only a small number of droplets left with a maximal diameter <1.0 μm (II of S8A Fig), and at 1:10, all droplets dissolved. Interestingly, for TssDNA, even at 1:1, many large droplets formed with a maximal diameter of approximately 4.0 μm (III of S8A Fig and S9 Video), while at 1:2, droplets were significantly dissolved, with only several droplets left with a maximal diameter <1.0 μm (IV of S8A Fig). At 1:5, all droplets were dissolved. T24 has a similar effect as TssDNA: at 1:1, many large droplets formed with a maximal diameter of approximately 5.0 μm (V of S8A Fig and S10 Video), while at 1:2, droplets were significantly dissolved, with several droplets left with a maximal diameter <1.0 μm (IV of S8A Fig). Strikingly, three oligonucleic acids also have the same two-stage effect on the FUS U-CTD (S8B Fig and S11, S12 and S13 Videos). These results together suggest that the folding of ZnF is not essential for the induction and dissolution of LLPS of the FUS CTD as induced by both ATP and oligonucleic acids.

We then monitored the two-stage effect of oligonucleic acids by HSQC titrations at the same molar ratios used for the above DIC imaging. For the F-CTD, upon adding TssDNA at 1:0.1, no significant shift of HSQC peaks was observed, but the peak intensity appeared to slightly reduce. At 1:0.5, there was still no shift of peaks, but the peak intensity reduced significantly (S7C and S7D Fig). At 1:1, all HSQC peaks disappeared, and further addition even up to 1:50 led to no reappearance of HSQC peaks. T24 also showed the same pattern for the F-CTD.

On the other hand, for the FUS U-CTD, addition of TssDNA at 0.1 and 0.5 led to no significant shift of HSQC peaks but only the reduction of the peak intensity, while at 1:1, all HSQC peaks also disappeared, which is similar to what was observed on the F-CTD. Further addition to 1:5, at which LLPS was completely dissolved, resulted in the reappearance of some HSQC peaks, and the reappearance became saturated at 1:10 (Fig 7B). T24 also had a similar effect, but only with the positions of some reappeared HSQC peaks slightly different from those induced by TssDNA (Fig 7C).

We subsequently calculated the CSD between the FUS U-CTD in the free state and in the presence of TssDNA or T24 at a molar ratio of 1:10 (Fig 7D). Most interestingly, both ssDNAs have very similar overall patterns of CSD, which are also similar to that induced by ATP. Briefly, most residues with significantly shifted HSQC peaks are 25 Arg and 4 Lys residues, strongly suggesting that ATP and ssDNA specifically bind to these Arg/Lys residues. Noticeably, out of three regions of the FUS CTD, RGG3 shows the most significant shifts, which is most likely due to the fact that it contains 15 Arg residues (approximately 21% of residues 454–256), while RGG2 only has 8 Arg residues, accounting for only 15% of residues 371–422. It is particularly worthwhile to notice that although the 2 Arg (6% of residues 422–454) and 3 Lys residues located within the unfolded ZnF have the neighboring sequences fundamentally different from those of RGG2 and RGG3, their HSQC peaks are also significantly shifted. This observation suggests that the binding of ATP/ssDNA to Arg/Lys residues is not highly dependent on the sequence hosting Arg/Lys residues as long as it is disordered. The results clearly indicate that Arg/Lys residues are key residues for both ATP and nucleic acids to target for both induction and dissolution of LLPS of the FUS CTD.

Therefore, a speculative model was proposed for the induction and dissolution of LLPS of both the F-CTD (S7E Fig) and U-CTD (Fig 7E) by ATP and nucleic acids. Briefly, at low concentrations, ATP can act as a bivalent binder to induce LLPS of both the F-CTD and U-CTD by using the aromatic ring of its adenine group bind to the side chains of Arg/Lys on the one hand and its triphosphate chain to bind Arg/Lys on the other. Consequently, large and dynamic ATP-CTD complexes will be formed, manifesting as liquid droplets. However, at high concentrations, the exceeding amount of ATP molecules will disrupt these large and dynamic complexes, thus leading to the dissolution of the droplets. Interestingly, because the FUS CTD contains no aromatic-residue–rich regions like the NTD that can further self-assemble into large oligomers upon being overbound with ATP (Fig 6D), HSQC peaks of the FUS CTD molecules overbound with ATP are still detectable. Nucleic acids also bind to Arg/Lys residues to induce and dissolve LLPS as ATP but have much higher affinity because of the ability to establish multivalent binding to the FUS CTD.

### ATP and oligonucleic acids enhance and then dissolve LLPS of FUS

Recently, we found that ATP enhanced LLPS of full-length FUS at low ATP concentrations, followed by dissolution of LLPS at high concentrations and characterized by gradual intensity reduction of all HSQC peaks [27]. Further detailed analysis revealed that the intensity reduction occurred uniformly for all residues of FUS. To understand this observation, here we characterized the effects of AMP and adenosine on LLPS of full-length FUS by DIC and NMR. The results showed that AMP was still able to induce the changes of HSQC peak intensity like ATP but was much less effective (S9A Fig). However, no significant dissolution of LLPS was observed with the AMP concentration even up to 10 mM, and only with the concentration to 50 mM could LLPS be dissolved. On the other hand, adenosine only had a very weak capacity to induce the reduction of HSQC peak intensity with its concentration up to 10 mM (S9B Fig), at which no detectable effect on LLPS of FUS was observed.

In parallel to DIC imaging (Fig 4), we also characterized the oligonucleic-acid–induced enhancement and dissolution of LLPS of full-length FUS by NMR. First, we superimposed the HSQC spectrum of full-length FUS with that of the isolated RRM (S10A Fig), CTD (S10B Fig), or NTD, respectively (S10C Fig), which were all collected under the same protein concentration and buffer conditions. The results revealed that while in full-length FUS, HSQC peaks of the disordered regions were all detectable and highly superimposable to those of the isolated domains, some HSQC peaks of the folded RRM and ZnF slightly shifted or disappeared in full-length FUS. This was only observed on full-length FUS, and in fact, all HSQC peaks of RRM could be detected in FUS (1–371), containing the NTD and RRM, as well as FUS (267–526), consisting of RRM and the entire CTD [41]. This implies that the conformations of different domains are highly similar in the isolated form and in full-length FUS, but in full-length FUS, additional interactions and/or μs–ms dynamics exist, likely resulting from dynamic intermolecular interactions underlying LLPS, thus leading to slight shifts and disappearance of some HSQC peaks of the well-folded RRM and ZnF domains. In general, the intensity of HSQC peaks of a folded protein is much weaker than that of an unfolded protein because T2 values of the residues in the well-folded state are much shorter than those in the unfolded state. For example, we found previously that for the coexisting folded and unfolded states of the ALS-causing C71G-Profilin (PFN) mutant [69], HSQC peaks of the unfolded state were much stronger than those of the folded state, although the population of the unfolded state was lower than that of the folded state.

**Fig 8:**
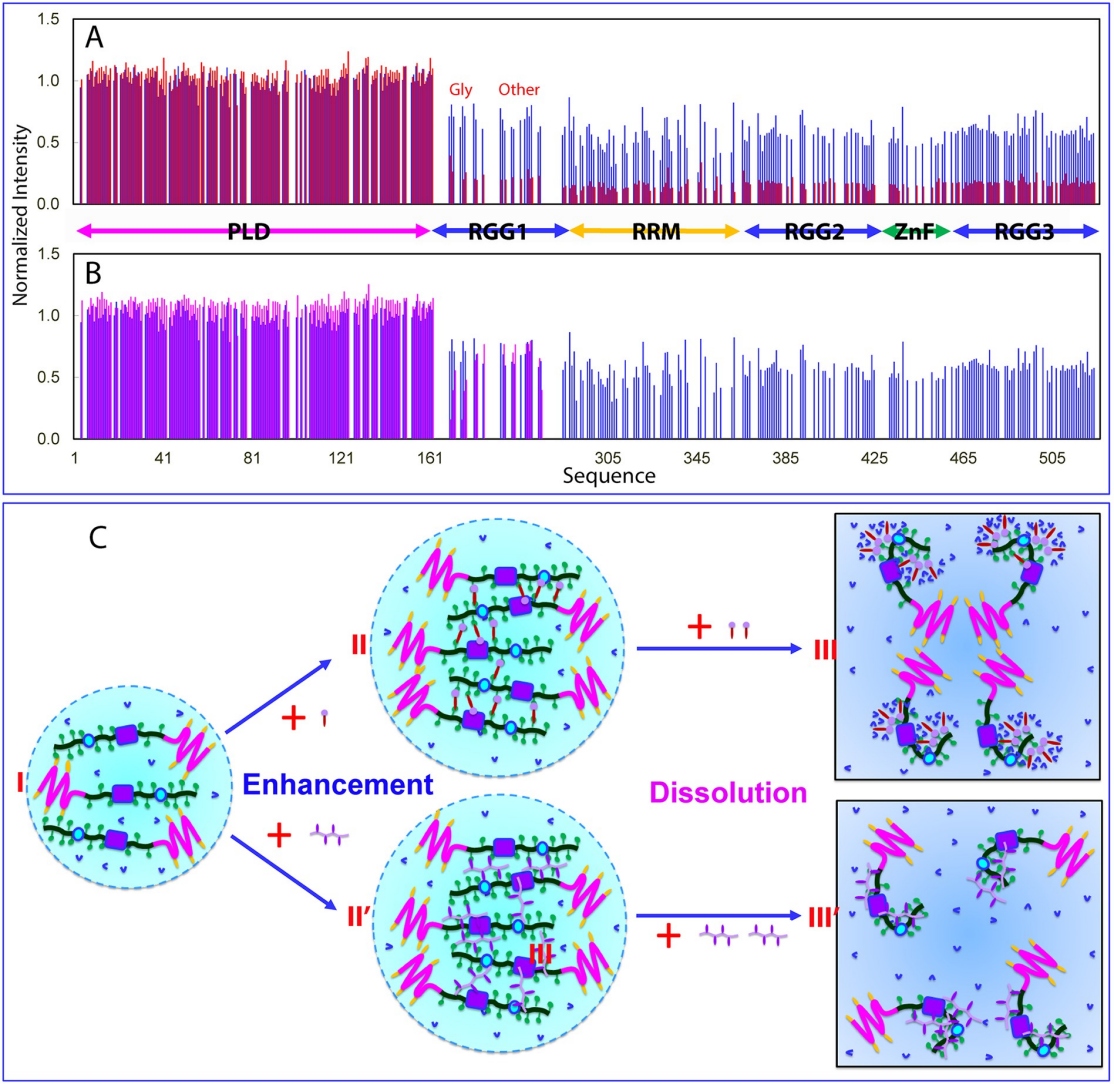
NMR view of enhancement and dissolution of LLPS of FUS induced by ATP and ssDNA. (A) Normalized HSQC peak intensity of the ^15^N-labeled FUS in the presence of TssDNA at molar ratios of 1: 0.1 (blue) and 1:0.5 (red) as divided by that of FUS in the free state. (B) Normalized HSQC peak intensity of the ^15^N-labeled FUS in the presence of TssDNA at molar ratios of 1: 0.1 (blue) and 1:10 (purple) as divided by that of FUS in the free state. (C) A speculative model to rationalize the specific binding of ATP and ssDNA to Arg/Lys residues as well as RRM and ZnF of FUS to enhance LLPS at low concentrations but dissolution at high concentrations. FUS, Fused in sarcoma; HSQC, Heteronuclear single quantum coherence spectroscopy; LLPS, liquid–liquid phase separation; PLD, prion-like domain; RGG, Arg-Gly/Arg-Gly-Gly–rich region; RRM, RNA-recognition motif; ssDNA, single-stranded DNA; TssDNA, telomeric ssDNA; ZnF, zinc finger.

We subsequently titrated full-length FUS with both TssDNA and T24 at different molar ratios, including 1:0.1, 1:0.5, 1:1, 1:2, 1:5, 1:10, :1:15, 1:20, 1:25, 1:30, 1:35, 1:40, 1:45, and 1:50. Briefly, two ssDNA molecules triggered highly similar changes of HSQC spectra of FUS. At low molar ratios, addition of TssDNA led to no significant shift of HSQC peaks but changes in peak intensity (S11A Fig). However, upon addition of TssDNA at a ratio of 1:1, under which LLPS was significantly enhanced (Fig 4), all HSQC peaks disappeared. Unexpectedly, when the ratio reached 1:5, some HSQC peaks stared to reappear, and the reappearance was completed at 1:10 (S11B Fig). Further addition of TssDNA up to 1:50 showed no further changes. Interestingly, the HSQC spectrum of FUS in the presence of TssDNA at 1:10 is highly superimposable to that of the FUS NTD in the presence of ssDNA alone at 1:5 (S11C Fig). Therefore, we analyzed the intensity of HSQC peaks of FUS without significant overlap in the absence and in the presence of TssDNA at different ratios. Very interestingly, the PLD residues have their intensity slightly increased at a ratio of 1:0.1 and more increased at 1:0.5 (Fig 8A). By contrast, the peak intensity of the RGG1, RRM, RGG2, ZnF, and RGG3 regions uniformly decreased (Fig 8B). Most strikingly, at 1:10, almost all peaks of RRM, RGG2, ZnF, and RGG3 residues remained undetectable, but the peaks of the PLD and some RGG1 residues reappeared (S11C Fig). The intensity of most PLD residues in the presence of ssDNA at 1:10 is even slightly higher than that in the free state (Fig 8B).

To test whether ATP and oligonucleic acids also share the same binding sites in the context of full-length FUS, we added ATP into an FUS sample to 3 mM, and consequently, the HSQC peak intensity was significantly reduced (S12A Fig). Subsequently, we gradually added in TssDNA or T24 at ratios of 1:0.1, 1:0.5, 1:1, 1:2.5, 1:5, 1:10, 1:15; 1:20; 1:25, and 1:50. Upon adding TssDNA at a ratio of 1:10, corresponding to 0.2 mM of TssDNA, many HSQC peaks reappeared, and the reappearance became complete at 1:15 (0.3 mM). Further addition up to 1:50 showed no significant changes (S12B Fig). Noticeably, the HSQC spectra of FUS in the presence of both ATP at 3 mM and TssDNA at 0.3 mM are highly superimposable to that of the FUS NTD in the presence of TssDNA alone at 1:5 (S12C Fig). This observation implies that 1) the residues of full-length FUS binding to ATP and TssDNA are the same or at least highly overlapped; and 2) the binding affinity of TssDNA to these residues is much higher than that of ATP, and consequently, TssDNA at 0.3 mM could displace ATP at 3 mM from binding with FUS.

Therefore, by binding to almost the same residues of the FUS molecule, ATP and oligonucleic acids achieve modulation of LLPS of the full-length FUS in the same manner: enhancement at low concentrations but dissolution at high concentrations (Fig 8C). Briefly, ATP is capable of bivalent binding to Arg/Lys residues by utilizing its adenine aromatic rings to establish the π-cation interaction with Arg/Lys and the phosphate oxyanion of its triphosphate group to establish electrostatic interactions with Arg/Lys residues. On the other hand, oligonucleic acids are most likely use their base aromatic rings and phosphate oxyanion to achieve multivalent binding to Arg/Lys residues by establishing both π-cation and electrostatic interactions. At low concentrations, ATP and oligonucleic acids will facilitate the formation of large and dynamic multivalent complexes of FUS, manifesting as the enhancement of LLPS. Because of the size and dynamics, NMR signals for both types of complexes are undetectable. However, at high concentrations, the exceeding number of ATP or oligonucleic acid molecules will disrupt the dynamic complexes, thus manifesting as dissolution of LLPS. However, because of the unique hydrotropic properties, overbinding of ATP to FUS will lead to the formation of the dynamic oligomers (III of Fig 8C). Consequently, the NMR signals of all FUS residues, including the PLD residues, are undetectable even though ATP has no significant binding to the PLD. By contrast, because ssDNA has no such hydrotropic properties, at the high concentrations of oligonucleic acids at which LLPS is dissolved, the binding of oligonucleic acids to Arg/Lys residues is largely saturated (III of Fig 8C). Consequently, the NMR signal of the LPD residues becomes detectable because the PLD residues have no significant binding to oligonucleic acids, and the motions of the disordered PLD residues are rather independent from the rest of the FUS molecule overbound with nucleic acids. Furthermore, the binding of oligonucleic acids with RGG1 containing only 9 Arg residues and 1 Lys appears to be more dynamic than the binding with RRM, RGG2, ZnF, and RGG3. Consequently, the HSQC peaks of some RGG1 residues also reappeared, but the intensity is lower than those of the PLD residues, which have no significant binding to nucleic acids.

## Discussion

Previously, LLPS of well-folded proteins such as lysozyme has been extensively characterized to occur only at very high concentrations (>mM) because they were mostly driven by nonspecific molecular interactions [14,38,39,70–72]. On the other hand, the key proteins for forming membraneless organelles are characterized by containing large intrinsically disordered domains/regions and can phase separate at very low concentrations (approximately μM) [12–17,35,40,73–75]. FUS contains two types of intrinsically disordered regions accounting for 75% of the sequence (Fig 1A): the PLD belongs to the prion-like family extensively found in RBPs [76,77], while three RG/RGG regions carry the “RG/RGG motif” identified in >1,700 human proteins [78–82]. Intrinsically disordered RGG regions constitute a nonclassic category capable of binding various nucleic acids [28,42,61,62,78–82]. Therefore, the FUS CTD containing both well-folded ZnF and RGG regions is an archetype for a large assay of the proteins with intrinsically disordered domains rich in Arg/Lys residues [78–82].

Here, we first assessed LLPS of FUS and its six differentially dissected domains. The results indicate that the isolated PLD containing 24 Tyr residues but lacking Arg/Lys failed to efficiently phase separate, thus revealing that the π–π interaction alone is relatively weak in driving LLPS. This is further supported by the results that both ATP and oligonucleic acids also failed to induce LLPS of the PLD. On the other hand, despite containing 5 Tyr and 5 Phe as well as 35 Arg and 4 Lys residues, the FUS CTD was also unable to phase separate regardless of being folded or unfolded for ZnF, thus implying that without other interactions, most proteins rich in RGG motifs are not able to phase separate by themselves. Indeed, the intrinsically disordered PGL-3 protein responsible for forming the P-granule, which has an Arg-rich C-terminus, only phase separates in the presence of mRNA [33]. Strikingly, despite having conformation and dynamics similar to those of PLD, the FUS NTD with the additional 9 Arg and 1 Lys residues was sufficient to effectively phase separate with a capacity comparable to that of full-length FUS, thus revealing that the π-cation interaction is the major driving force for LLPS of FUS, completely consistent with the systematic mutagenesis studies [40]. Because the FUS NTD is an independent unit sufficient for initiating liposarcoma development even by separate expression with CHOP [18–20], this finding might offer a clue for future in vivo investigation to assess whether LLPS is also involved in its pathological activity.

Subsequently, we successfully defined the mechanism for ATP and nucleic acids to modulate LLPS of FUS. We found that ATP and oligonucleic acids not only modulate LLPS of full-length FUS but also its dissected domains in the same manner. Interestingly, although ATP and oligonucleic acids manifested different NMR observations upon interacting with FUS, both of them were revealed to modulate LLPS by primarily binding to Arg/Lys residues, as evidenced by the residue-specific NMR results. However, oligonucleic acids have a much higher capacity than ATP in binding with FUS, which most likely results from their ability to establish dynamic but multiple-site binding to the Arg/Lys residues within the disordered regions through both π-cation and electrostatic interactions [35,40,51,52,83].

Very importantly, we found that regardless of being folded or unfolded for ZnF, FUS CTD could be induced to phase separate at low concentrations followed by dissolution at high concentrations by both ATP and oligonucleic acids through specific binding to the Arg/Lys residues, which even include those Arg/Lys residues located within the unfolded ZnF domain without “RG/RGG” sequence motifs. Furthermore, in the context of full-length FUS, oligonucleic acids bind all domains except for the PLD and are also able to displace ATP from binding to FUS. This strong ability for nucleic acids to displace ATP may bear significant implications in understanding the interplay between ATP and nucleic acids in modulating the physiological and pathological roles of FUS in cells. In the cellular environments where nucleic acids are available at high concentrations, ATP is expected to be largely displaced from binding with the disordered regions, and consequently, FUS is predominantly bound with nucleic acids with LLPS dissolved. As a result, its PLD will be available for functioning as a transcriptional activation domain by binding to various partners, which include the CTD of RNA polymerase II and hormone and retinoid receptors [3,18–20,84–86].

Our results together reveal that unlike binding to the well-folded domains, which requires the specific pockets constituted by different residues [87,88], out of 20 residues within the disordered regions, ATP and nucleic acids have the most significant interactions with Arg/Lys residues, which are not necessarily hosted by “RG/RGG” sequence motifs or coexisting with the folded domains such as RRM and ZnF capable of binding nucleic acids. Indeed, we have also studied the interactions of the 152-residue TDP-43 PLD [30] with ATP and oligonucleic acids and found that they only significantly perturbed 5 Arg and 1 Lys residues, out of which only one is hosted by the “RG” sequence. Moreover, recent reports suggest that disordered “Arg-Ser (RS) motifs” also play a key role in binding nucleic acids [81,82].

Recently, the ALS-causing C9orf72 dipeptide repeats extremely rich in Arg have been shown to impair the assembly, dynamics, and function of SGs [89–91]. Our results here suggest that the ability for C9orf72 dipeptide repeats to perturb SGs may at least partly result from their binding with the aromatic residues in the low-complexity regions of the protein components such as FUS and/or with nucleic acids within SGs. Indeed, very recently, the C9orf72 dipeptide repeats have been identified to manifest their cellular toxicity by becoming tightly associated with any accessible nucleic acids in cells and consequently to impair all reactions of these nucleic acids [92]. This finding is also supported by the observation that protamine, a sperm-specific small polypeptide with the highest percentage of Arg/Lys content within the animal proteome [93], also manifests similar cytotoxicity as C9orf72 repeats by tight binding to nucleic acids [92,93].

In consideration of the general involvement of nucleic acids in forming various membraneless organelles and very high concentrations of ATP in all living cells, in the future, it is of fundamental and physiological significance to understand the interplay of ATP and nucleic acids in binding and/or modulation of LLPS of a myriad of proteins with intrinsically disordered regions rich in Arg/Lys residues. In fact, previously, it has been already revealed that ATP determined the viscosity of the droplets formed by RNA and proteins in the oocyte nucleolus [94,95]. Our current results suggest that in addition to providing energy to drive a variety of active processes regulating the viscosity, ATP might also be able to modulate the viscosity of many membraneless organelles by direct binding to Arg/Lys residues.

In conclusion, our study indicates that LLPS of FUS appears to be primarily driven by the π-cation interaction between aromatic and Arg/Lys residues, which also serve as the target for modulation by ATP and nucleic acids through specific binding. Specific and multiple-site interactions among intrinsically disordered regions in driving LLPS provide a mechanism for the observation that unlike the well-folded proteins, proteins such as FUS with intrinsically disordered regions can phase separate at very low concentrations. Furthermore, one possible reason for the fact that only a small number of organelle types are observed in eukaryotic cells might result from the specificity of the interactions driving LLPS as well as the specific and direct modulation of LLPS in cells by ATP and nucleic acids. Noticeably, many pathological factors appear to manifest their cytotoxicity by hacking into this mechanism, and consequently, to safeguard this mechanism might represent a promising avenue to develop therapeutic strategies/molecules.

## Materials and methods

### Preparation of recombinant FUS protein and nucleic acid samples

Previously, we have cloned DNA fragments encoding FUS and its differentially dissected fragments into a modified vector pET28a with a C-terminal His-tag [41]. Although the presence of 6×His-tag had no detectable effect on their solution conformation, we found that the presence of His-tag has slightly weakened their capacity in LLPS. Therefore, in the present study, we removed the His-tag by adding a stop codon immediately after the DNA sequences encoding full-length FUS; FUS (1–165) containing the PLD; FUS (1–267) composed of the PLD and RGG1; FUS (1–371) consisting of the PLD, RGG1, and RMM; FUS (267–526) composed of RRM and the CTD; and FUS (371–526) consisting of RGG2, ZnF, and RGG3 (Fig 1A). The expression and purification of these recombinant FUS proteins followed the previously established protocols in which RP-HPLC purification was used to remove any possible association of cellular nucleic acids with FUS proteins [41].

To generate isotope-labeled proteins for NMR studies, the same procedures were used, except that the bacteria were grown in M9 medium with addition of (^15^NH_4_)_2_SO_4_ for ^15^N-labeling and (^15^NH_4_)_2_SO_4_/^13^C-glucose for ^15^N-/^13^C-double labeling [41]. The protein concentration was determined by the UV spectroscopic method in the presence of 8 M urea, under which the extinct coefficient at 280 nm of a protein can be calculated by adding up the contribution of Trp, Tyr, and Cys residues [41,96].

ATP, AMP, and adenosine were purchased from Sigma-Aldrich (St. Louis, MO, USA) with the same catalog numbers as previously reported [26,27]. MgCl_2_ was added into ATP for stabilization by forming the ATP-Mg complex [26,27]. The 12-mer RNA with a sequence of UAGUUUGGUGAU was purchased from Integrated DNA Technologies Pte. Ltd. (Coralville, IA, USA), while the 24-mer ssDNA with a sequence of (TTAGGG)_4_ and T_24_ were purchased from a local company/branch of Integrated DNA Technologies Pte. Ltd. (Singapore, Singapore) [31]. The protein and ssDNA samples were all prepared in 5 mM sodium phosphate buffer containing 1 mM MgCl_2_, and the final solution pH was adjusted to 6.0 with aliquots of very diluted NaOH or HCl.

### CD experiments

CD characterization of TssDNA and T24 was performed on a Jasco J-1500 spectropolarimeter (Jasco, Tokyo, Japan) equipped with a thermal controller as previously described [41]. The spectra were collected in a 10-mm cuvette at TssDNA and T24 concentrations of 10 μM in different buffers at pH 6.0, as indicated in S4 Fig.

### Optimization of experimental conditions

Although in cells, it was estimated that FUS has a concentration of approximately 12 μM [32], and we found that in our in vitro system, FUS at a saturation concentration of 2 μM was sufficient to significantly phase separate, here we used 20 μM because at lower concentrations, the NMR signals are too weak to obtain high-quality spectra for analysis. For pH, here we used a near-neutral value of 6.0 because LLPS of FUS and its domains showed no significant difference in LLPS at pH 6.0 and 6.8, but unfortunately, at pH values higher than 6.0, many NMR signals became undetectable (S1D Fig). Furthermore, high salt concentrations would significantly reduce the enhancing effect for NMR signal sensitivity by the cryoprobe, which, however, is essential for NMR HSQC titration experiments at such a low protein concentration (20 μM).

We have characterized the effect of three oligonucleic acids (one RNA and two ssDNA molecules) on LLPS of FUS and its domains by measuring the turbidity at 600 nm in parallel with monitoring formation of droplets by DIC microscopy. We found that turbidity value is much more dependent on the size than the number of the droplets: while the solution containing a large number of droplets with diameters <1 μm had negligible absorption values at 600 nm, the solution containing only a few droplets but with diameters >5 μm could have very high absorption values. This is likely due to the fact that the ability for droplets to reflect and scatter light is highly dependent on their diameters. Therefore, we further attempted to obtain a quantitative measurement of the droplet formation and dissolution by picking up all droplets and measured their diameters by the software built in the microscopy. However, as for the small droplets with diameters <1 μm, there are more than one layer of droplets, and consequently, it is extremely challenging to precisely pick up all droplets in the view field. To the best of our knowledge, currently there is no other better method to quantify LLPS without need of labeling with external reagents. As for our current study, we just need a qualitative estimation of enhancement and dissolution of LLPS at different ratios of nucleic acids. Therefore, to avoid misleading, we only presented semiquantitative descriptions of DIC imaging and also provided the key video files in Supporting Information.

### DIC microscopy

The formation of liquid droplets was imaged at 25 °C on 50 μl of different FUS samples at a protein concentration of 20 μM in 5 mM sodium phosphate buffer at pH 6.0 containing 1 mM MgCl_2_ in the absence and in the presence of ATP-Mg complex at 0.1, 0.5, 1, 2, 3, 4, 5, 6, 7, 8, 9, and 10 mM, as previously conducted [27]; in the presence of AMP at 1, 2, 3, 4, 5, 6, 7, 8, 9, 10, 20, 30, 40, and 50 mM; in the presence of adenosine at 1, 2, 3, 4, 5, 6, 7, 8, 9, and 10 mM; and RNA or ssDNA at different molar ratios (FUS:RNA/ssDNA) including 1:0.1, 1:0.5, 1:1, 1:2, 1:5, 1:10, :1:15, 1:20, 1:25, 1:30, 1:35, 1:40, 1:45, and 1:50 by DIC microscopy (OLYMPUS IX73 Inverted Microscope System with OLYMPUS DP74 Color Camera; Olympus, Tokyo, Japan) as previously described [27,31]. ZnCl_2_ was also added to the sample of full-length FUS and the CTD with the folded ZnF to reach a final concentration of 4 mM.

### NMR characterizations

All NMR experiments were acquired at 25 °C on an 800 MHz Bruker Avance spectrometer (Bruker, Billerica, MA, USA) equipped with pulse field gradient units and a shielded cryoprobe as described previously [27,41,46,97]. To have the enhancing effect of the cryoprobe for NMR signal sensitivity, which is essential for NMR HSQC titration experiments at a very low protein concentration (20 μM), NMR samples had to be prepared in 5 mM sodium phosphate buffer while pH value was optimized to 6.0 because many HSQC peaks of highly disordered NTDs and CTDs disappeared at higher pH values. ZnCl_2_ was also added to the sample of full-length FUS and the CTD with the folded ZnF to reach a final concentration of 4 mM.

For achieving sequential assignments of the FUS CTD with ZnF folded or unfolded, NMR three-dimensional experiments including cross-bond triple-resonance experiments HN(CO)CACB, CBCA(CO)NH, CCC(CO)NH, and HNN were acquired on a ^15^N-/^13^C-double labeled sample, while cross-space ^15^N-edited HSQC-TOCSY and HSQC-NOESY were collected on ^15^N-labeled samples of the FUS CTD with ZnF either folded or unfolded at a protein concentration of 500 μM in 5 mM sodium phosphate buffer at pH 6.0 [97]. NMR ^1^H chemical shifts were referenced to external DSS at 0.0 ppm. NH, N, Hα, Cα, and Cβ chemical shifts of FUS CTDs were further analyzed by SSP program [53] to gain quantitative insights into the populations of different secondary structures. NMR data were processed with NMRPipe [98] and analyzed with NMRView [99].

For NMR titration studies of the interactions between FUS/domains and ATP/ssDNA, one-dimensional proton and two-dimensional ^1^H-^15^N NMR HSQC spectra were collected on ^15^N-labeled samples at a protein concentration of 20 μM in 5 mM sodium phosphate buffer (pH 6.0) containing 1 mM MgCl_2_ at 25 °C in the absence and in the presence of ATP, AMP, and adenosine, as well as two ssDNA molecules at concentrations or molar ratios exactly as those used for the above DIC imaging.

To probe the interactions of FUS and its domains with ATP and two ssDNA molecules by NMR line broadening, the peak intensity of HSQC spectra was respectively normalized by dividing the intensity with that of a reference HSQC spectrum of FUS/domains in the free state at the same protein concentration (20 μM) and buffer conditions (5 mM sodium phosphate at pH 6.0) as previously reported [31]. To calculate the CSD induced by interactions with ATP and two ssDNA molecules, the HSQC spectra were superimposed, and subsequently, the shifted peaks were identified and further assigned to the corresponding residues. The degree of perturbation was reported by an integrated index calculated by the following formula [22,100]:

$${\left({\left(\Delta^{1}\text{H}\right)}^{2}+\;{\left(\Delta^{15}\text{N}\right)}^{2}/5\right)}^{1/2}.$$ (1)

### NMR ^15^N backbone dynamics of the FUS NTD on a ps–ns timescale

^15^N backbone T1 and T1ρ relaxation times and {^1^H}-^15^N steady-state NOE intensities were collected on a ^15^N-labeled FUS (1–267) at 25 °C at protein concentration of 50 μM in 5 mM sodium phosphate buffer (pH 6.0) on an Avance 800 MHz Bruker spectrometer with both an actively shielded cryoprobe and pulse field gradient units [41,43,44,46,47]. Relaxation time T1 was determined by collecting 7 points with delays of 10, 160, 400, 500, 640, 800, and 1,000 ms using a recycle delay of 1 s, with a repeat at 400 ms. Relaxation time T1ρ was measured by collecting 8 points with delays of 1, 40, 80, 120, 160, 200, 240, and 280 ms, with a repeat at 120 ms. {^1^H}-^15^N steady-state NOEs were obtained by recording spectra with and without ^1^H presaturation, a duration of 3 s, and a relaxation delay of 6 s at 800 MHz.

The ^15^N backbone relaxation data of FUS (1–267) were analyzed by direct mapping of the reduced spectral density with simplified approximations [44,47]. Briefly, J(0), J(ωN), and J(0.87ωH), the spectral densities at the frequencies 0, ωN, and 0.87ωH, respectively, were calculated based on the following equations:

$$\delta_{\text{NH}}{=\;\text{R}}_{\text{1}}{\text{(NOE-1)}\gamma }_{\text{N}}{/\gamma }_{\text{H}}\text{,}$$ (2)

$$\text{J}\left(0\right)=\;(6{\text{R}}_{2}\;-\;3{\text{R}}_{1}\;-\;2.72\delta_{\text{NH}})/\left(3{\text{d}}^{2}+\;4{\text{c}}^{2}\right),$$ (3)

$$\text{J}(\omega \text{N})=\;(4{\text{R}}_{1}\;-\;5\delta_{\text{NH}})/\left(3{\text{d}}^{2}+\;4{\text{c}}^{2}\right),$$ (4)

$$\text{J}(0.87\omega \text{H})=\;4\delta_{\text{NH}}/\left(5{\text{d}}^{2}\right),$$ (5)

where d = (μ_0_hγ_N_ γ_H_/8π^2^)(r ^-3^) and c = ωNΔδ/3^1/2^.

### NMR ^15^N backbone dynamics on a μs–ms timescale

^15^N transverse relaxation dispersion experiments were acquired at 25 °C on the ^15^N-labeled FUS (1–267) on a Bruker Avance 800 spectrometer [41,45,46]. A constant time delay (*T*_CP_ = 50 ms) was used with a series of CPMG frequencies, ranging from 40 Hz, 80 Hz, 120 Hz (×2), 160 Hz, 200 Hz, 240 Hz, 320 Hz, 400 Hz, 480 Hz, 560 Hz, 640 Hz, 720 Hz, 800 Hz, and 960 Hz (×2 indicates repetition). A reference spectrum without the CPMG block was acquired to calculate the effective transverse relaxation rate by the following equation:

$$R_{2}^{eff}=-\text{ln}\left(I\left(v_{CPMG}\right)/I_{0}\right)/T_{CP,}$$ (6)

where I(ν_CPMG_) is the peak intensity on the difference CPMG frequency and I_0_ is the peak intensity in the reference spectra.

### PGF diffusion measurement

PGF-NMR experiments were run on an 800 MHz Bruker Avance spectrometer at 25 °C. The different protein samples were prepared in D_2_O at protein concentration of 50 μM in 1 mM sodium phosphate buffer (pD 6.0). The experiments were performed using the Bruker pulse sequence and the Bruker macro diffusion ordered spectroscopy (DOSY) [48,50]. Typically, 16 values of gradient strength were used in the range 0 to 32 G/cm, with PFG duration of 2 ms and diffusion time of 150 ms. The self-diffusion coefficients (*D*_*s*_) were calculated using the Bruker DOSY analysis program. Each sample was run in triplicate, and *Ds* values were averaged over the three experiments. The resulting decay curves were fitted, and *Ds* values were calculated with the equation below:

$$\text{I}\;=\;\text{I}\left(0\right){\text{exp}[-\text{D}(\gamma \text{g}\delta )}^{\text{2}}(\text{Δ}\;-\;(\text{δ}/3))],$$ (7)

where I(0) is 1.002, γ is 4.258 x 10^3^ Hz/G, δ is 4.000 ms, and Δ is 150 ms.

## Supporting information

S1 Data NMR chemical shifts of the FUS CTD with the folded and unfolded ZnF. CTD, C-terminal domain; FUS, Fused in sarcoma; ZnF, zinc finger.
(XLSX)

S1 Fig Compositions, LLPS, and conformations of FUS and its domains. (A) Amino-acid compositions of the FUS PLD (1–165), RGG1 (166–271), and CTD (371–526). DIC microscopy images of liquid droplets formed by FUS (B) and NTD (C). (D) HSQC spectra of the ^15^N-labeled NTD (1–267) (blue) and PLD (1–165) (red) at 20 μM in 5 mM sodium phosphate buffer at pH 4.0 and 6.8. CTD, C-terminal domain; DIC, differential interference contrast; FUS, Fused in sarcoma; HSQC, Heteronuclear single quantum coherence spectroscopy; LLPS, liquid–liquid phase separation; NTD, N-terminal domain; PLD, prion-like domain; RGG1, RG/RGG-rich region 1.
(TIF)

S2 Fig ^15^N NMR backbone relaxation data of the FUS NTD. ^15^N NMR backbone relaxation data of the FUS NTD as measured at 800 MHz. (A) {^1^H}-^15^N steady-state NOE intensities. (B) R1. (C) R2. FUS, Fused in sarcoma; NOE, Nuclear Overhauser Effect Spectroscopy; NTD, N-terminal domain.
(TIF)

S3 Fig Residue-specific conformations of the FUS F-CTD and U-CTD. (A) Amino-acid sequence of the FUS CTD with the ZnF residues underlined. Arg residues are colored in blue and Lys in green. (B) HSQC spectra of the ^15^N-labeled FUS CTD with the assignment of the folded ZnF residues labeled. Residue-specific values of the FUS CTD with the unfolded (blue) or folded (red) ZnF for (ΔCα–ΔCβ) (C), (ΔHα) (D), and SSP (E). CTD, C-terminal domain; FUS, Fused in sarcoma; F-CTD, ZnF folded CTD; HSQC, Heteronuclear single quantum coherence spectroscopy; SSP, Secondary Structure Propensity; U-CTD, ZnF unfolded CTD; ZnF, zinc finger.
(TIF)

S4 Fig CD characterization of solution conformations of TssDNA and T24. CD spectra over 200–340 nm collected in different buffers at pH 6.0 for T24 (A); and TssDNA (B). CD, circular dichroism; ssDNA, single-stranded DNA; TssDNA, telomeric ssDNA.
(TIF)

S5 Fig NMR view of interactions of NTD/PLD with ATP, AMP, adenosine, and ssDNA. HSQC spectra of the ^15^N-labeled FUS NTD in the absence (blue) and in the presence of AMP (A) and adenosine (B) at 10 mM, respectively. HSQC spectra of the ^15^N-labeled FUS PLD in the absence (blue) and in the presence of ATP at 10 mM (C) and TssDNA at a ratio of 1:50 (D). AMP, Adenosine monophosphate; FUS, Fused in sarcoma; HSQC, Heteronuclear single quantum coherence spectroscopy; NTD, N-terminal domain; PLD, prion-like domain; ssDNA, single-stranded DNA; TssDNA, telomeric ssDNA.
(TIF)

S6 Fig DIC imaging of LLPS of the FUS NTD induced by RNA and ssDNA. DIC images of liquid droplets formed by the FUS NTD in the absence (A) and in the presence of RNA (B); TssDNA (C) and T24 (D) at different molar ratios. DIC, differential interference contrast; FUS, Fused in sarcoma; LLPS, liquid–liquid phase separation; NTD, N-terminal domain; ssDNA, single-stranded DNA; TssDNA, telomeric ssDNA.
(TIF)

S7 Fig NMR View of interactions of the FUS F-CTD with AMP, adenosine, and ssDNA. HSQC spectra of the ^15^N-labeled FUS F-CTD in the absence and in the presence of AMP (A), adenosine (B), and TssDNA (C) at different molar ratios. (D) Normalized HSQC peak intensity of the ^15^N-labeled FUS F-CTD in the presence of TssDNA at molar ratios of 1: 0.1 (blue) and 1:0.5 (red) as divided by that in the free state. (E) A speculative model to rationalize the specific binding of ATP and ssDNA to Arg/Lys residues within the FUS F-CTD to induce LLPS at low concentrations but to dissolve at high concentrations. AMP, Adenosine monophosphate; CTD, C-terminal domain; FUS, Fused in sarcoma; F-CTD, ZnF folded CTD; HSQC, Heteronuclear single quantum coherence spectroscopy; LLPS, liquid–liquid phase separation; ssDNA, single-stranded DNA; TssDNA, telomeric ssDNA; ZnF, zinc finger.
(TIF)

S8 Fig DIC imaging of the induction and dissolution of LLPS of FUS CTD as mediated by ssDNA. (A) DIC microscopy images of liquid droplets formed by the FUS F-CTD in the presence of RNA and two ssDNA molecules at different molar ratios. (B) DIC microscopy images of liquid droplets formed by FUS U-CTD in the presence of RNA and two ssDNA molecules at different molar ratios. The videos for outputting these images are provided in Supporting Information. CTD, C-terminal domain; DIC, differential interference contrast; FUS, Fused in sarcoma; F-CTD, ZnF folded CTD; LLPS, liquid–liquid phase separation; ssDNA, single-stranded DNA; U-CTD, ZnF unfolded CTD; ZnF, zinc finger.
(TIF)

S9 Fig NMR view of interactions of FUS with AMP and adenosine. HSQC spectra of the ^15^N-labeled FUS in the absence and in the presence of AMP (A) and adenosine (B) at different concentrations. AMP, Adenosine monophosphate; FUS, Fused in sarcoma; HSQC, Heteronuclear single quantum coherence spectroscopy.
(TIF)

S10 Fig NMR view of the solution conformation of the full-length FUS. Superimposition of HSQC spectrum of the full-length FUS (blue) with that of RRM (red) (A), that of the F-CTD (red) (B), and that of the NTD (red) (C). CTD, C-terminal domain; FUS, Fused in sarcoma; F-CTD, ZnF folded CTD; HSQC, Heteronuclear single quantum coherence spectroscopy; NTD, N-terminal domain; RRM, RNA-recognition motif; ZnF, zinc finger.
(TIF)

S11 Fig NMR view of ssDNA-induced enhancement and dissolution of LLPS of FUS. HSQC spectra of FUS in the absence (blue) and in the presence of TssDNA (red) at 1:0.5 (A); and at 1:10 (B). (C) HSQC spectra of the ^15^N-labeled FUS in the presence of TssDNA at 1:10 (red) and the FUS NTD (1–267) in the presence of TssDNA at 1:5 (blue). FUS, Fused in sarcoma; HSQC, Heteronuclear single quantum coherence spectroscopy; LLPS, liquid–liquid phase separation; NTD, N-terminal domain; ssDNA, single-stranded DNA; TssDNA, telomeric ssDNA.
(TIF)

S12 Fig Oligonucleic acids can displace ATP from binding to FUS. HSQC spectra of FUS in the absence (blue) and in the presence of ATP at 3 mM (red). (B) HSQC spectra of the full-length FUS in the absence (blue) and in the presence of ATP at 3 mM with an extra addition of TssDNA at a ratio of 1:15 (red). (C) HSQC spectra of the ^15^N-labeled FUS in the presence of ATP at 3 mM and TssDNA at a ratio of 1:15 (red) and the FUS NTD (1–267) in the presence of TssDNA at 1:5 (blue). FUS, Fused in sarcoma; HSQC, Heteronuclear single quantum coherence spectroscopy; NTD, N-terminal domain; ssDNA, single-stranded DNA; TssDNA, telomeric ssDNA.
(TIF)

S1 Video DIC imaging of the liquid droplets formed by the full-length FUS. DIC, differential interference contrast; FUS, Fused in sarcoma.
(WMV)

S2 Video DIC imaging of the liquid droplets formed by the full-length FUS in the presence of RNA at a molar ratio of 1:2. DIC, differential interference contrast; FUS, Fused in sarcoma.
(WMV)

S3 Video DIC imaging of the liquid droplets formed by the full-length FUS in the presence of RNA at a molar ratio of 1:10. DIC, differential interference contrast; FUS, Fused in sarcoma.
(WMV)

S4 Video DIC imaging of the liquid droplets formed by the full-length FUS in the presence of TssDNA at a molar ratio of 1:1. DIC, differential interference contrast; FUS, Fused in sarcoma; ssDNA, single-stranded DNA; TssDNA, telomeric ssDNA.
(WMV)

S5 Video DIC imaging of the liquid droplets formed by the full-length FUS in the presence of TssDNA at a molar ratio of 1:5. DIC, differential interference contrast; FUS, Fused in sarcoma; ssDNA, single-stranded DNA; TssDNA, telomeric ssDNA.
(WMV)

S6 Video DIC imaging of the liquid droplets formed by the full-length FUS in the presence of T24 at a molar ratio of 1:1. DIC, differential interference contrast; FUS, Fused in sarcoma.
(WMV)

S7 Video DIC imaging of the liquid droplets formed by the full-length FUS in the presence of T24 at a molar ratio of 1:5. DIC, differential interference contrast; FUS, Fused in sarcoma.
(WMV)

S8 Video DIC imaging of the liquid droplets formed by the FUS CTD with the folded ZnF in the presence of RNA at 1:2. CTD, C-terminal domain; DIC, differential interference contrast; FUS, Fused in sarcoma; ZnF, zinc finger.
(WMV)

S9 Video DIC imaging of the liquid droplets formed by the FUS CTD with the folded ZnF in the presence of TssDNA at 1:1. CTD, C-terminal domain; DIC, differential interference contrast; FUS, Fused in sarcoma; ssDNA, single-stranded DNA; TssDNA, telomeric ssDNA; ZnF, zinc finger.
(WMV)

S10 Video DIC imaging of the liquid droplets formed by the FUS CTD with the folded ZnF in the presence of T24 at 1:1. CTD, C-terminal domain; DIC, differential interference contrast; FUS, Fused in sarcoma; ZnF, zinc finger.
(WMV)

S11 Video DIC imaging of the liquid droplets formed by the FUS CTD with the unfolded ZnF in the presence of RNA at 1:2. CTD, C-terminal domain; DIC, differential interference contrast; FUS, Fused in sarcoma; ZnF, zinc finger.
(WMV)

S12 Video DIC imaging of the liquid droplets formed by the FUS CTD with the unfolded ZnF in the presence of TssDNA at 1:1. CTD, C-terminal domain; DIC, differential interference contrast; FUS, Fused in sarcoma; ssDNA, single-stranded DNA; TssDNA, telomeric ssDNA; ZnF, zinc finger.
(WMV)

S13 Video DIC imaging of the liquid droplets formed by the FUS CTD with the unfolded ZnF in the presence of T24 at 1:1. CTD, C-terminal domain; DIC, differential interference contrast; FUS, Fused in sarcoma; ZnF, zinc finger.
(WMV)

## Abbreviations

ALS: Amyotrophic lateral sclerosis
AMP: Adenosine monophosphate
CD: circular dichroism
CHOP: C/EBP Homologous protein
CPMG: Carr–Purcell–Meiboom–Gill
CSI: chemical shift index
CSD: chemical shift difference
CTD: C-terminal domain
DIC: differential interference contrast
DOSY: diffusion ordered spectroscopy
FET: FUS/TLS, EWS, and TAF15
FTLD: frontotemporal lobar dementia
FUS: Fused in sarcoma
F-CTD: ZnF folded CTD
GFP: green fluorescent protein
hNOE: [^1^H]-^15^N steady-state heteronuclear NOE
hnRNPA1: heterogeneous nuclear ribonucleoprotein A1
HSQC: Heteronuclear single quantum coherence spectroscopy
LC: low sequence complexity
LLPS: liquid–liquid phase separation
NLS: nuclear localization signal
NOESY: Nuclear Overhauser Effect Spectroscopy
NTD: N-terminal domain
PDB: Protein Data Bank
PFN: Profilin
PLD: prion-like domain
QGSY: Gln-Gly-Ser-Tyr
RBP: RNA-binding protein
RG: Arg-Gly
RGG: Arg-Gly-Gly
RP-HPLC: Reverse-phase high-performance liquid chromatography
RRM: RNA-recognition motif
RS: Arg-Ser
SG: stress granule
ssDNA: single-stranded DNA
SSP: Secondary Structure Propensity
TDP: TAR DNA-binding protein
TLS: Translocated in Sarcoma
TOCSY: Total Correlation Spectroscopy
TssDNA: telomeric ssDNA
U-CTD: ZnF unfolded CTD
ZnF: zinc finger
