# Supplementary figures and images for: A unified mechanism for LLPS of ALS/FTLD-causing FUS as well as its modulation by ATP and oligonucleic acids
Source: PLoS Biol. 2019 Jun 12;17(6):e3000327. doi: 10.1371/journal.pbio.3000327 (PMC6590835; doi:10.1371/journal.pbio.3000327)

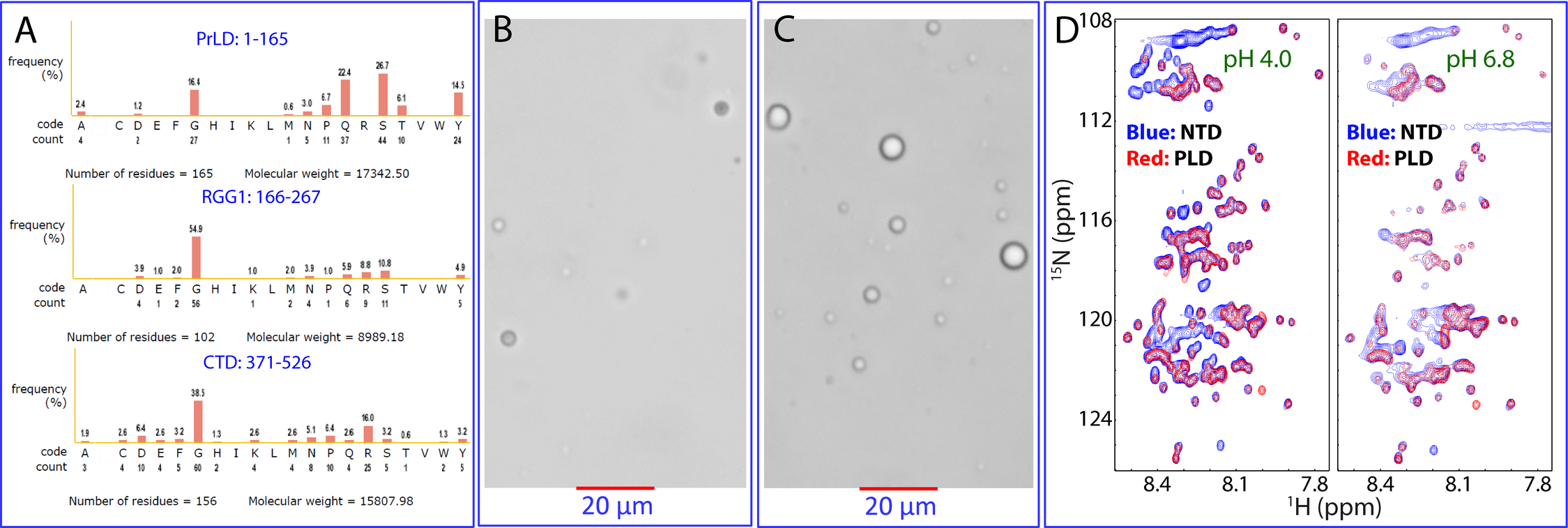

Supplement: S1 Fig — (A) Amino-acid compositions of the FUS PLD (1–165), RGG1 (166–271), and CTD (371–526). DIC microscopy images of liquid droplets formed by FUS (B) and NTD (C). (D) HSQC spectra of the 15N-labeled NTD (1–267) (blue) and PLD (1–165) (red) at 20 μM in 5 mM sodium phosphate buffer at pH 4.0 and 6.8. CTD, C-terminal domain; DIC, differential interference contrast; FUS, Fused in sarcoma; HSQC, Heteronuclear single quantum coherence spectroscopy; LLPS, liquid–liquid phase separation; NTD, N-terminal domain; PLD, prion-like domain; RGG1, RG/RGG-rich region 1. (TIF) [file pbio.3000327.s002.tif]

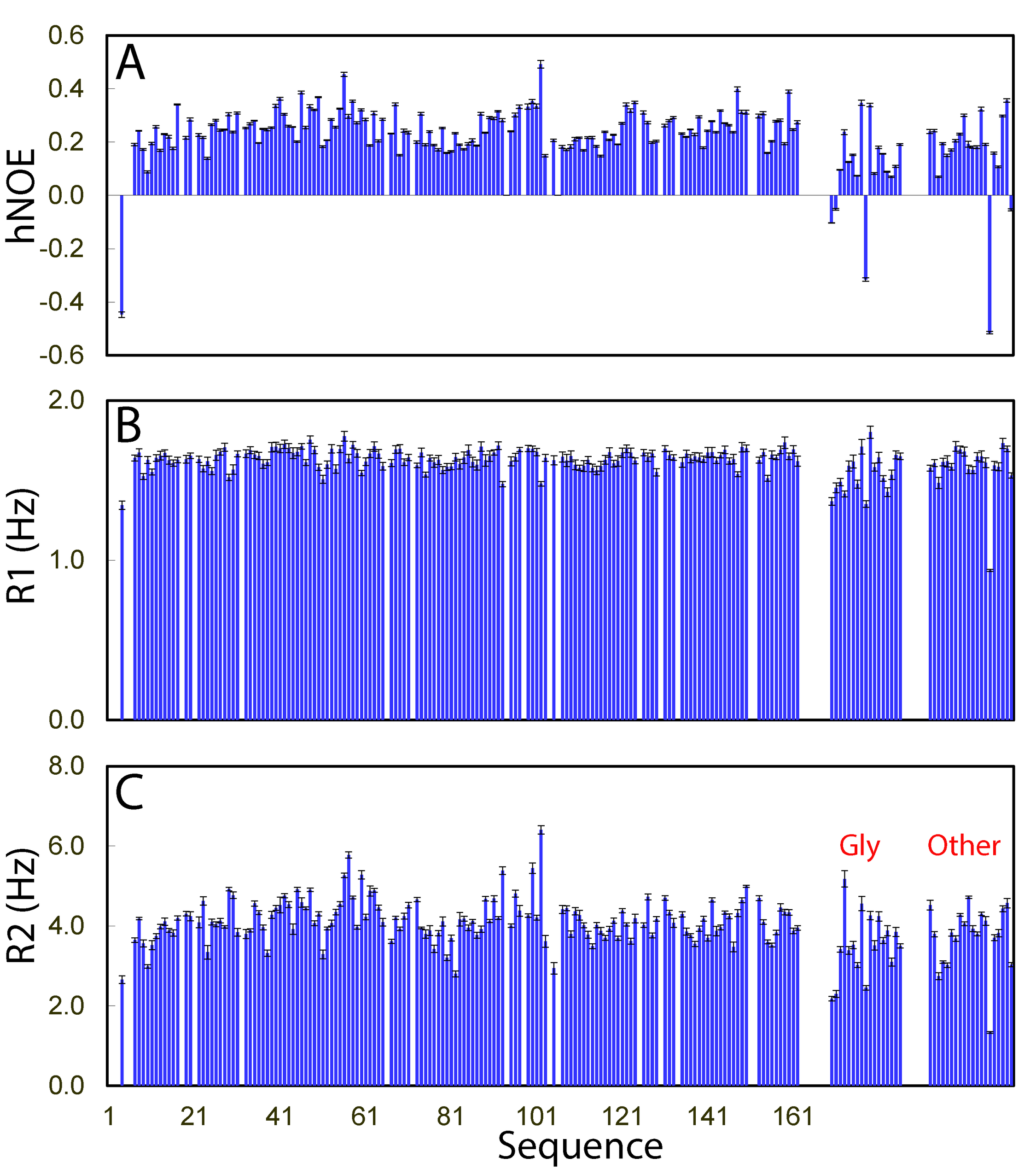

Supplement: S2 Fig — 15N NMR backbone relaxation data of the FUS NTD as measured at 800 MHz. (A) {1H}-15N steady-state NOE intensities. (B) R1. (C) R2. FUS, Fused in sarcoma; NOE, Nuclear Overhauser Effect Spectroscopy; NTD, N-terminal domain. (TIF) [file pbio.3000327.s003.tif]

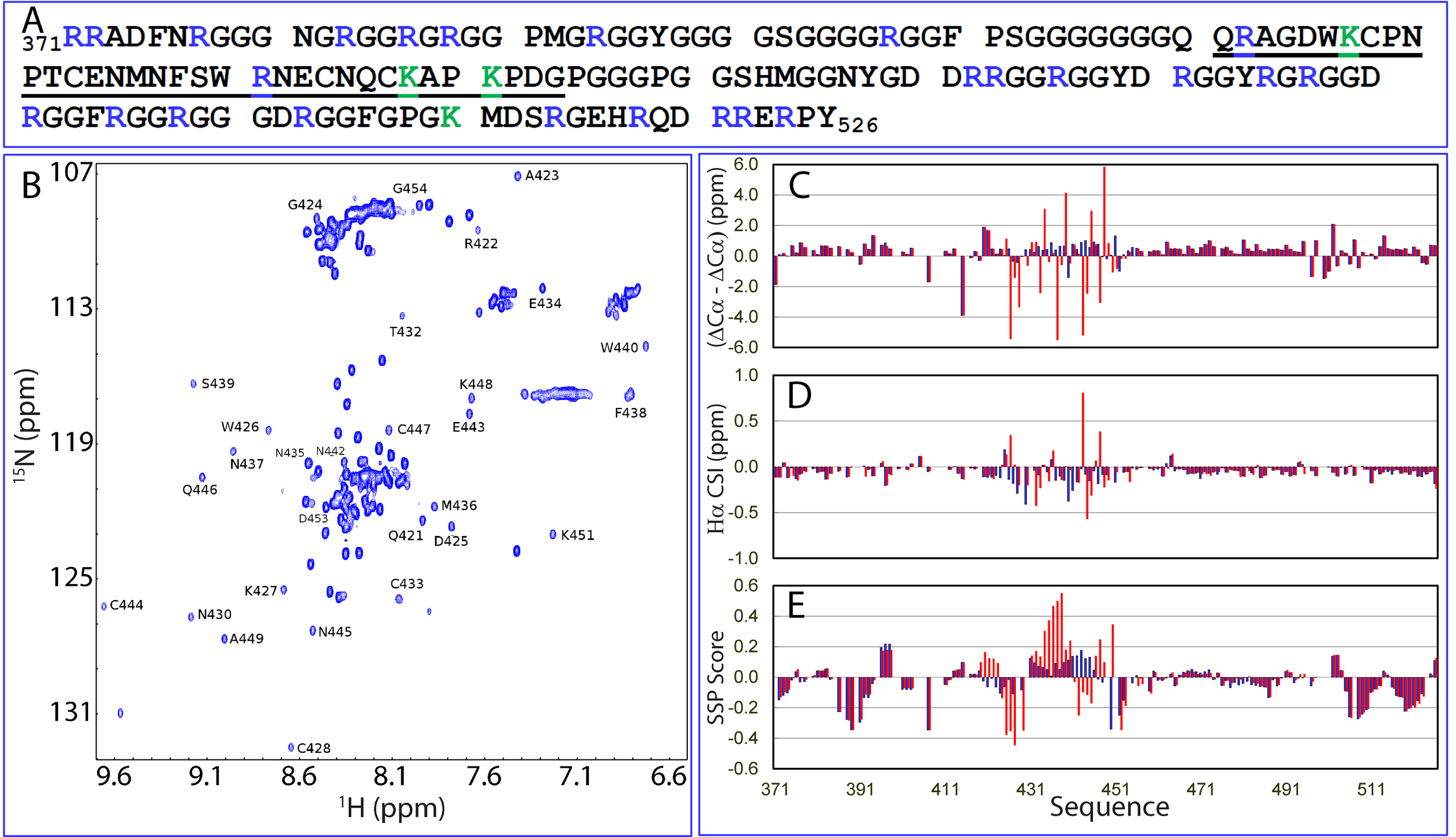

Supplement: S3 Fig — (A) Amino-acid sequence of the FUS CTD with the ZnF residues underlined. Arg residues are colored in blue and Lys in green. (B) HSQC spectra of the 15N-labeled FUS CTD with the assignment of the folded ZnF residues labeled. Residue-specific values of the FUS CTD with the unfolded (blue) or folded (red) ZnF for (ΔCα–ΔCβ) (C), (ΔHα) (D), and SSP (E). CTD, C-terminal domain; FUS, Fused in sarcoma; F-CTD, ZnF folded CTD; HSQC, Heteronuclear single quantum coherence spectroscopy; SSP, Secondary Structure Propensity; U-CTD, ZnF unfolded CTD; ZnF, zinc finger. (TIF) [file pbio.3000327.s004.tif]

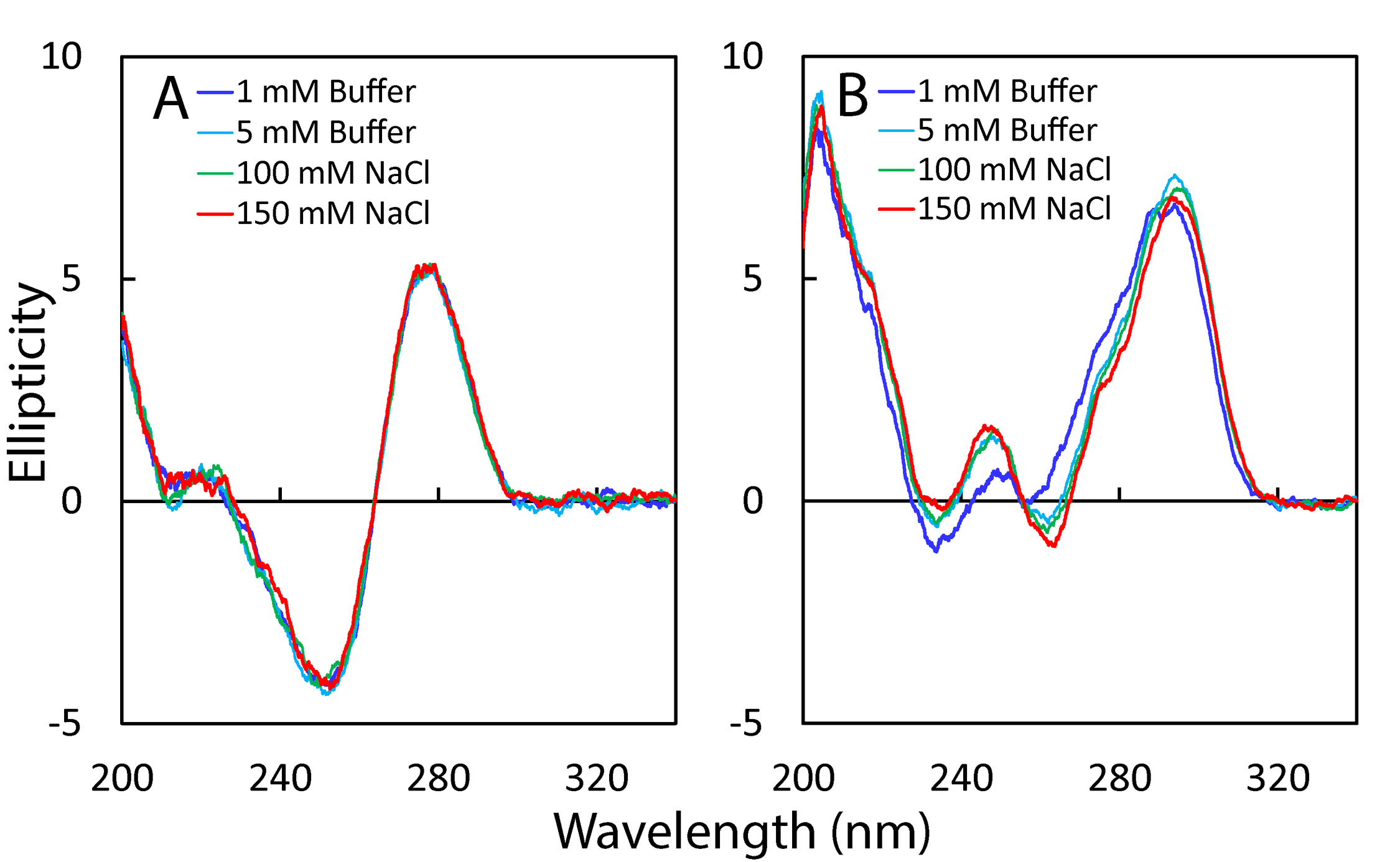

Supplement: S4 Fig — CD spectra over 200–340 nm collected in different buffers at pH 6.0 for T24 (A); and TssDNA (B). CD, circular dichroism; ssDNA, single-stranded DNA; TssDNA, telomeric ssDNA. (TIF) [file pbio.3000327.s005.tif]

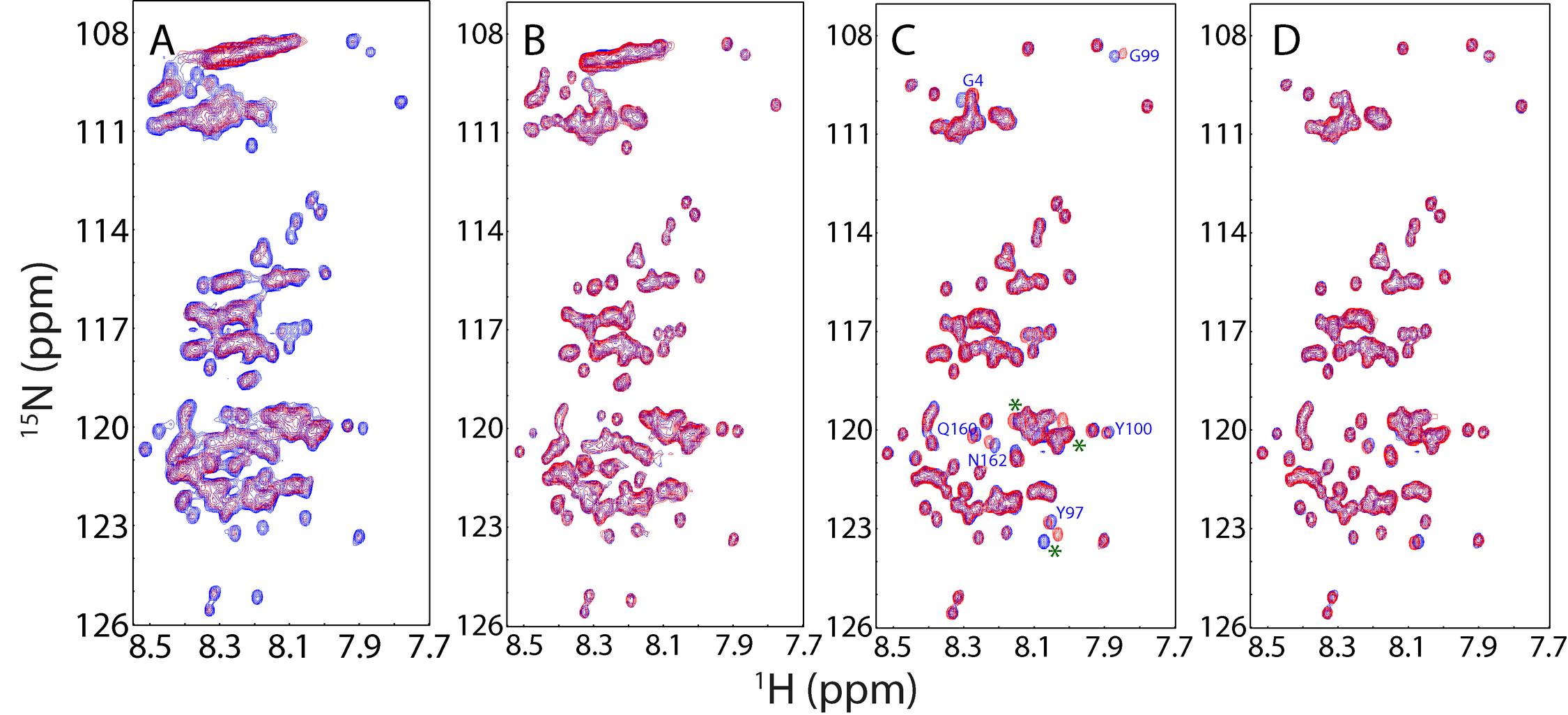

Supplement: S5 Fig — HSQC spectra of the 15N-labeled FUS NTD in the absence (blue) and in the presence of AMP (A) and adenosine (B) at 10 mM, respectively. HSQC spectra of the 15N-labeled FUS PLD in the absence (blue) and in the presence of ATP at 10 mM (C) and TssDNA at a ratio of 1:50 (D). AMP, Adenosine monophosphate; FUS, Fused in sarcoma; HSQC, Heteronuclear single quantum coherence spectroscopy; NTD, N-terminal domain; PLD, prion-like domain; ssDNA, single-stranded DNA; TssDNA, telomeric ssDNA. (TIF) [file pbio.3000327.s006.tif]

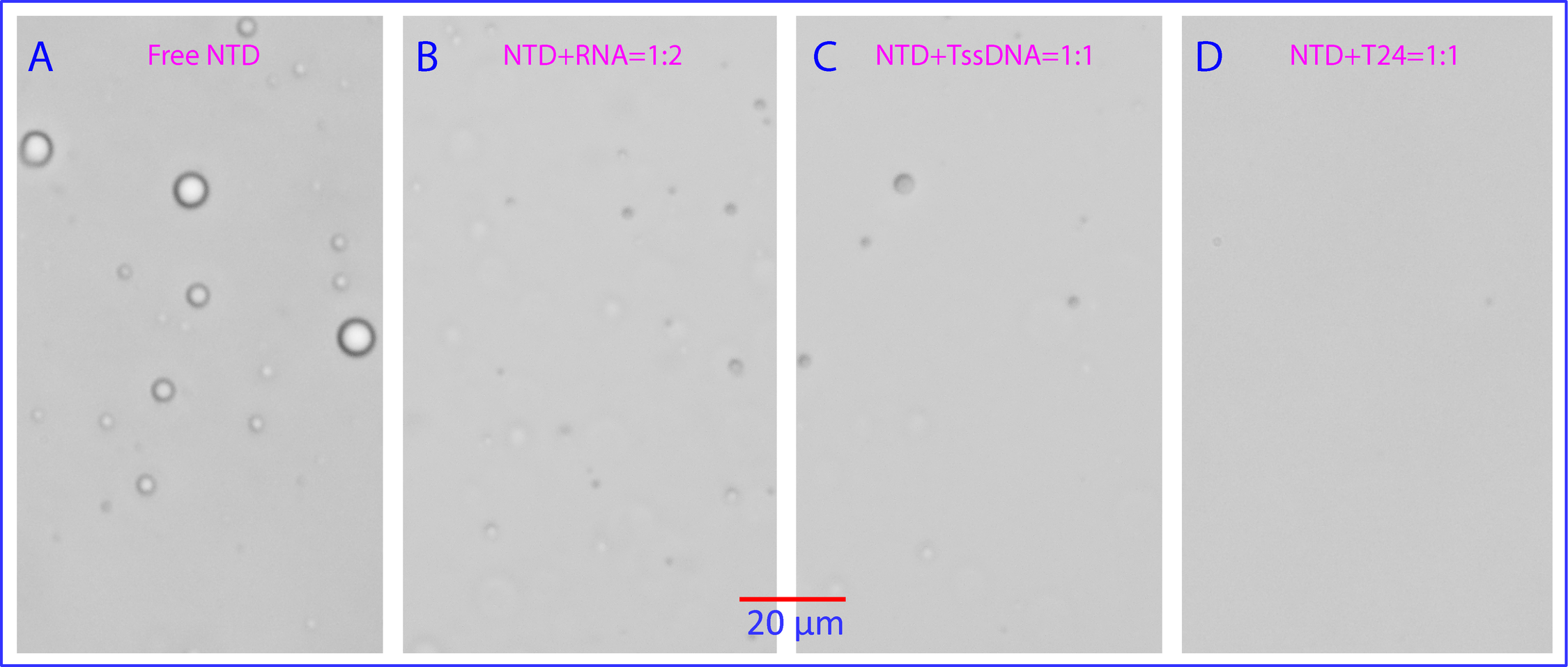

Supplement: S6 Fig — DIC images of liquid droplets formed by the FUS NTD in the absence (A) and in the presence of RNA (B); TssDNA (C) and T24 (D) at different molar ratios. DIC, differential interference contrast; FUS, Fused in sarcoma; LLPS, liquid–liquid phase separation; NTD, N-terminal domain; ssDNA, single-stranded DNA; TssDNA, telomeric ssDNA. (TIF) [file pbio.3000327.s007.tif]

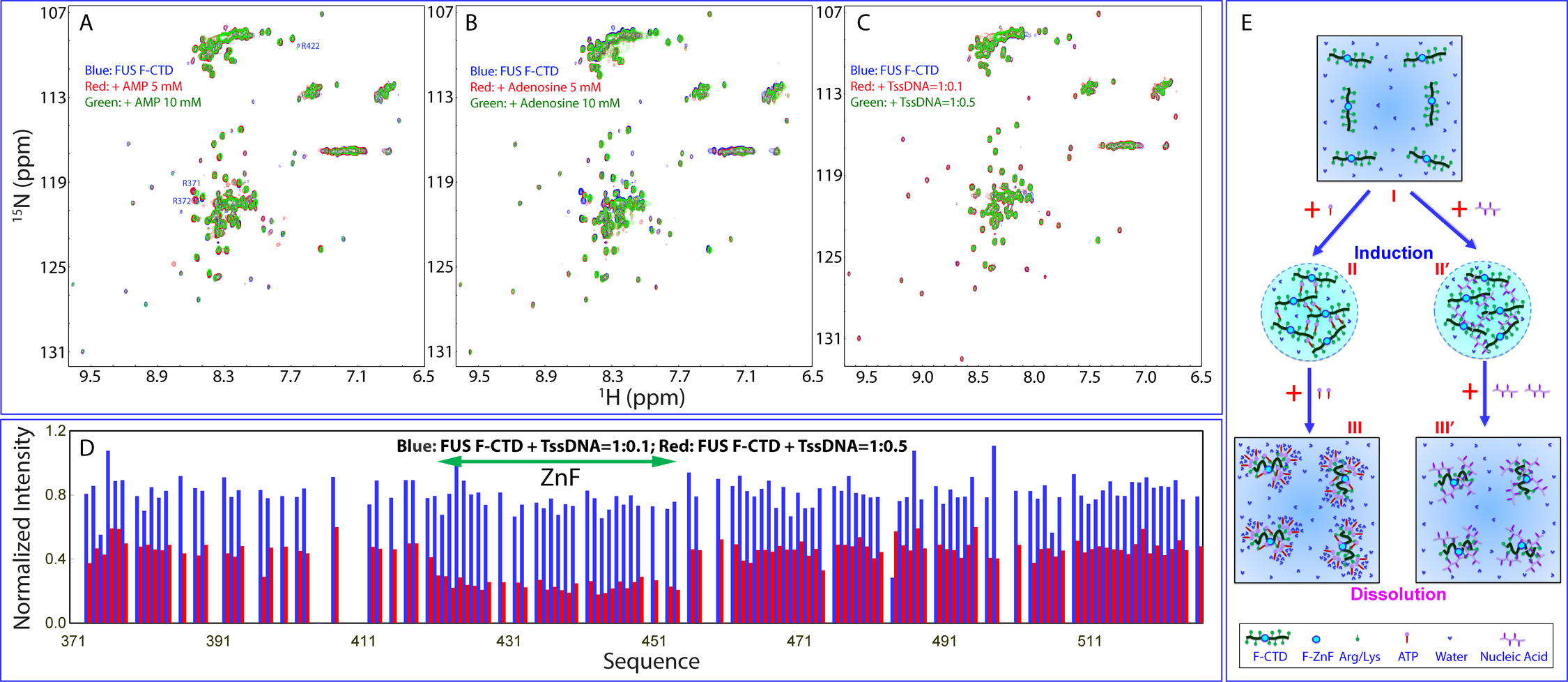

Supplement: S7 Fig — HSQC spectra of the 15N-labeled FUS F-CTD in the absence and in the presence of AMP (A), adenosine (B), and TssDNA (C) at different molar ratios. (D) Normalized HSQC peak intensity of the 15N-labeled FUS F-CTD in the presence of TssDNA at molar ratios of 1: 0.1 (blue) and 1:0.5 (red) as divided by that in the free state. (E) A speculative model to rationalize the specific binding of ATP and ssDNA to Arg/Lys residues within the FUS F-CTD to induce LLPS at low concentrations but to dissolve at high concentrations. AMP, Adenosine monophosphate; CTD, C-terminal domain; FUS, Fused in sarcoma; F-CTD, ZnF folded CTD; HSQC, Heteronuclear single quantum coherence spectroscopy; LLPS, liquid–liquid phase separation; ssDNA, single-stranded DNA; TssDNA, telomeric ssDNA; ZnF, zinc finger. (TIF) [file pbio.3000327.s008.tif]

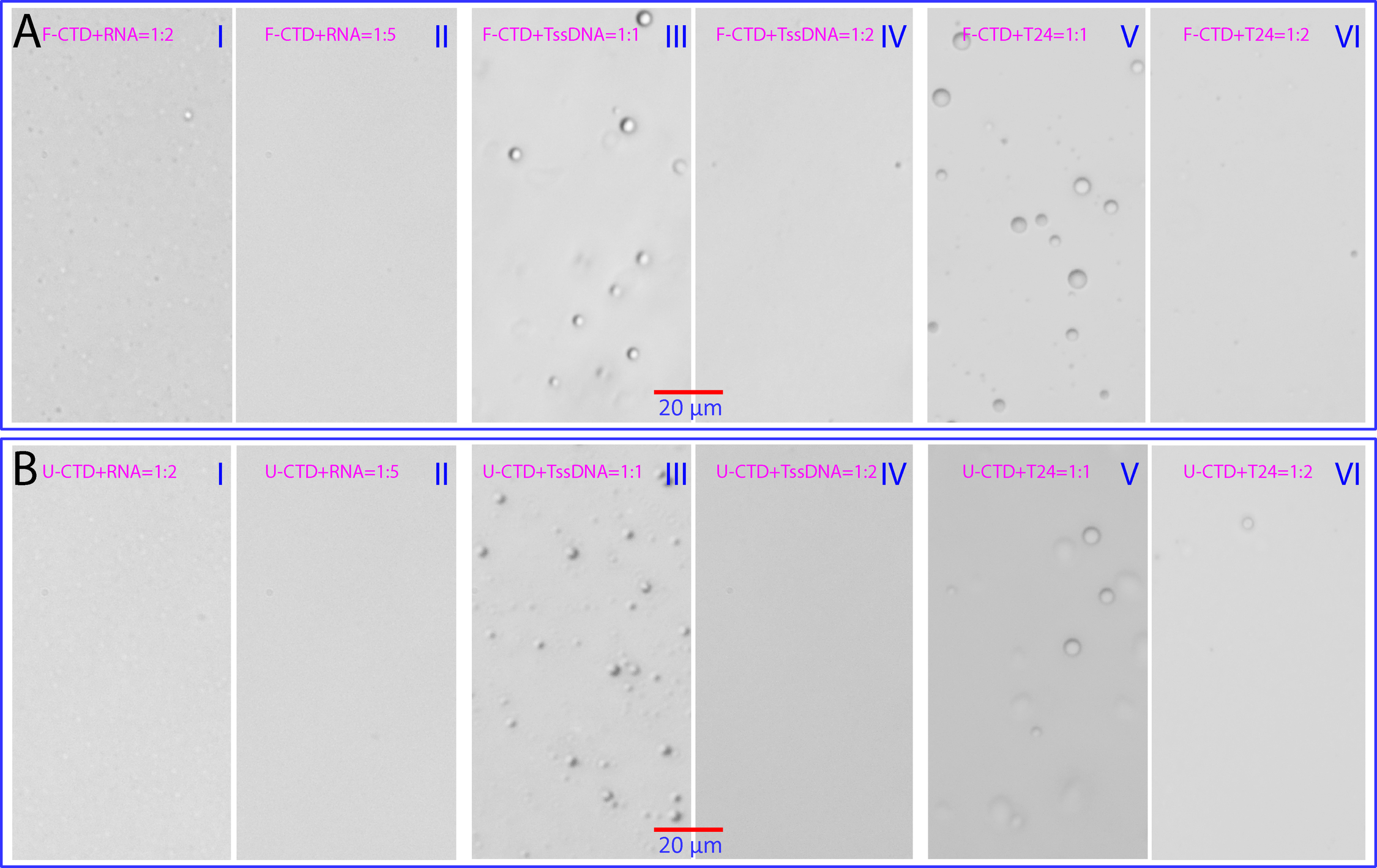

Supplement: S8 Fig — (A) DIC microscopy images of liquid droplets formed by the FUS F-CTD in the presence of RNA and two ssDNA molecules at different molar ratios. (B) DIC microscopy images of liquid droplets formed by FUS U-CTD in the presence of RNA and two ssDNA molecules at different molar ratios. The videos for outputting these images are provided in Supporting Information. CTD, C-terminal domain; DIC, differential interference contrast; FUS, Fused in sarcoma; F-CTD, ZnF folded CTD; LLPS, liquid–liquid phase separation; ssDNA, single-stranded DNA; U-CTD, ZnF unfolded CTD; ZnF, zinc finger. (TIF) [file pbio.3000327.s009.tif]

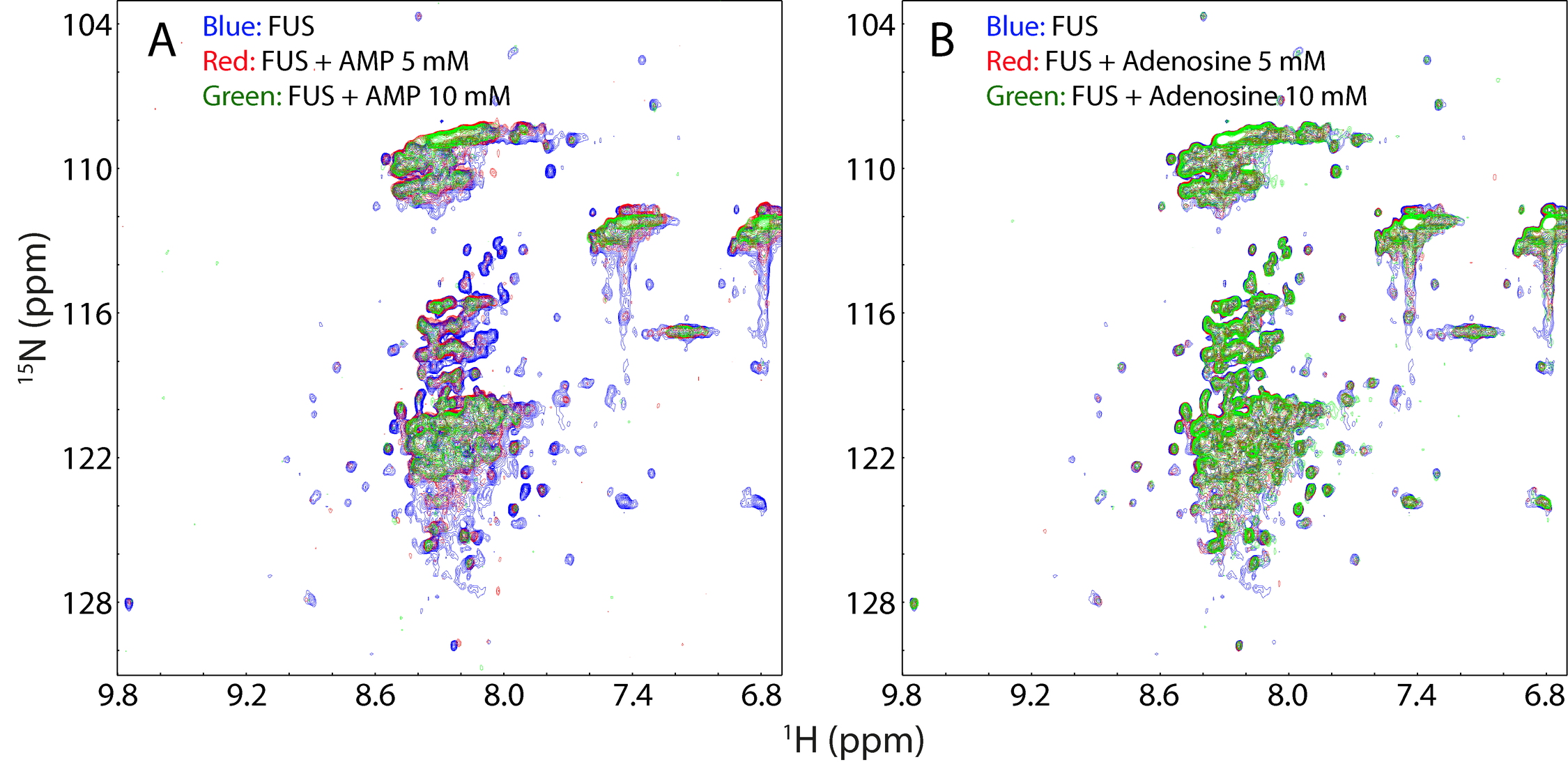

Supplement: S9 Fig — HSQC spectra of the 15N-labeled FUS in the absence and in the presence of AMP (A) and adenosine (B) at different concentrations. AMP, Adenosine monophosphate; FUS, Fused in sarcoma; HSQC, Heteronuclear single quantum coherence spectroscopy. (TIF) [file pbio.3000327.s010.tif]

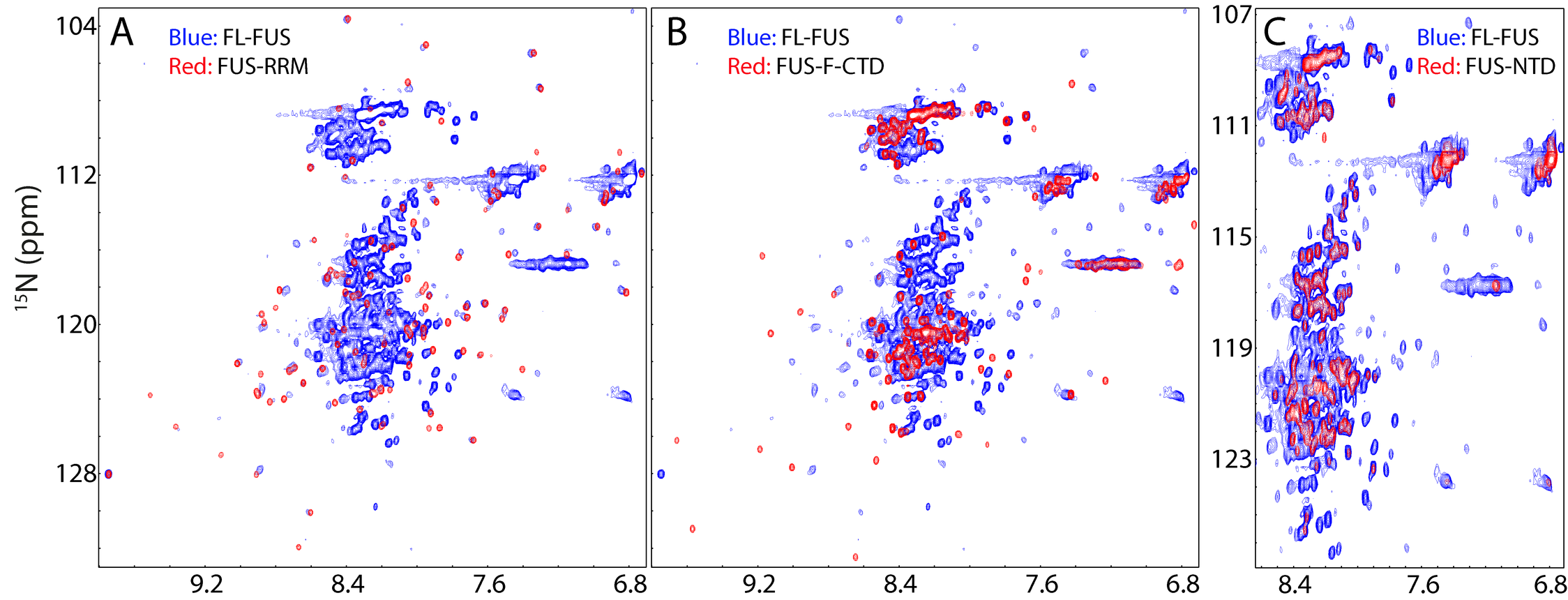

Supplement: S10 Fig — Superimposition of HSQC spectrum of the full-length FUS (blue) with that of RRM (red) (A), that of the F-CTD (red) (B), and that of the NTD (red) (C). CTD, C-terminal domain; FUS, Fused in sarcoma; F-CTD, ZnF folded CTD; HSQC, Heteronuclear single quantum coherence spectroscopy; NTD, N-terminal domain; RRM, RNA-recognition motif; ZnF, zinc finger. (TIF) [file pbio.3000327.s011.tif]

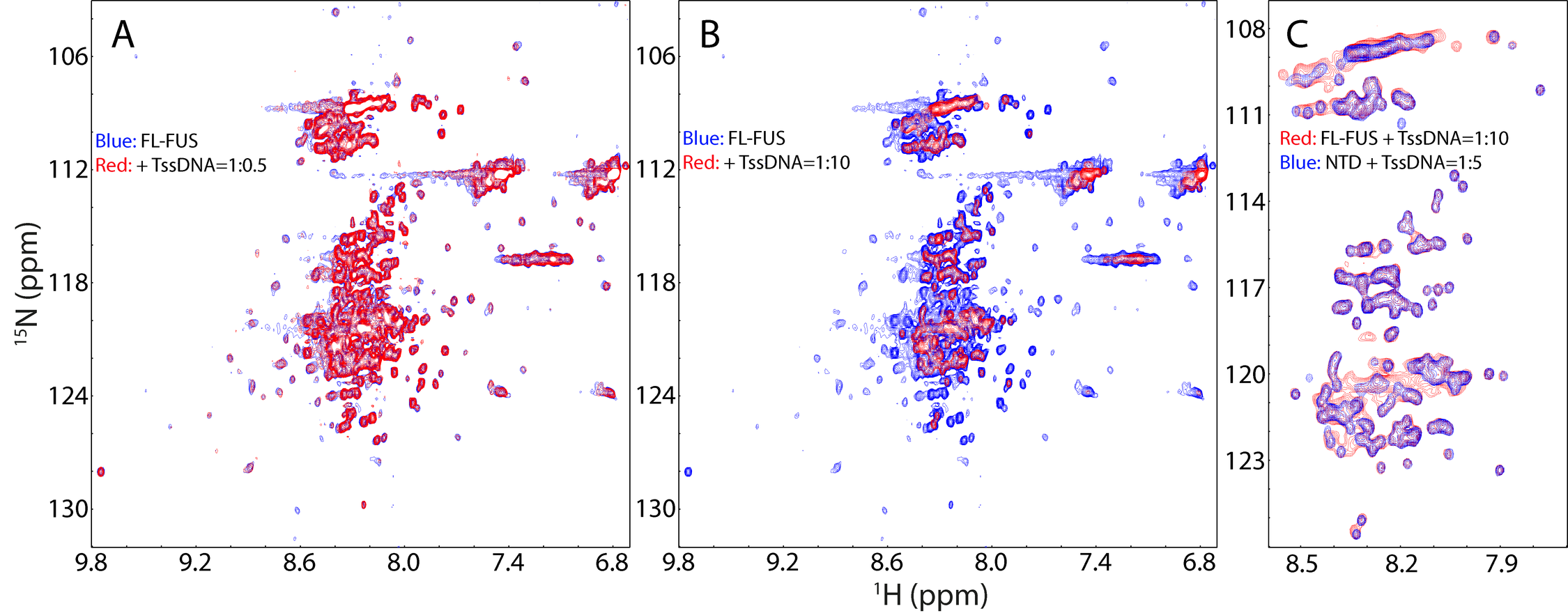

Supplement: S11 Fig — HSQC spectra of FUS in the absence (blue) and in the presence of TssDNA (red) at 1:0.5 (A); and at 1:10 (B). (C) HSQC spectra of the 15N-labeled FUS in the presence of TssDNA at 1:10 (red) and the FUS NTD (1–267) in the presence of TssDNA at 1:5 (blue). FUS, Fused in sarcoma; HSQC, Heteronuclear single quantum coherence spectroscopy; LLPS, liquid–liquid phase separation; NTD, N-terminal domain; ssDNA, single-stranded DNA; TssDNA, telomeric ssDNA. (TIF) [file pbio.3000327.s012.tif]

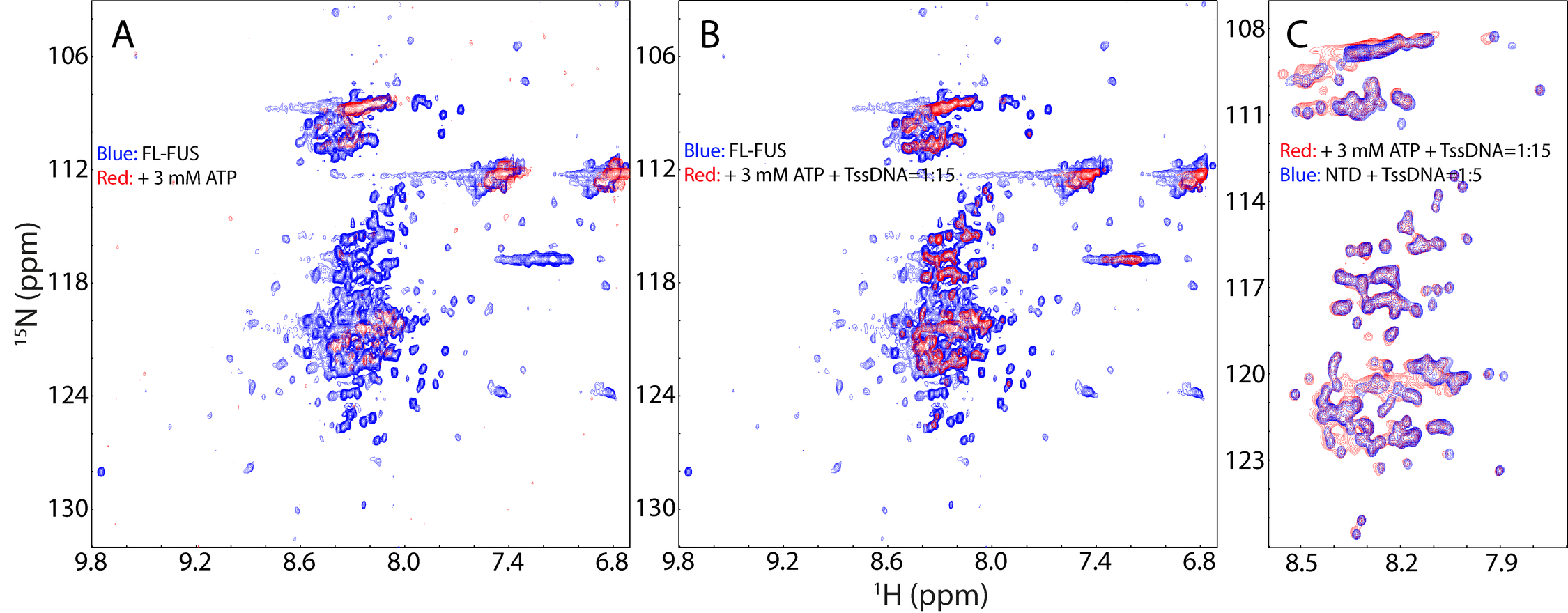

Supplement: S12 Fig — HSQC spectra of FUS in the absence (blue) and in the presence of ATP at 3 mM (red). (B) HSQC spectra of the full-length FUS in the absence (blue) and in the presence of ATP at 3 mM with an extra addition of TssDNA at a ratio of 1:15 (red). (C) HSQC spectra of the 15N-labeled FUS in the presence of ATP at 3 mM and TssDNA at a ratio of 1:15 (red) and the FUS NTD (1–267) in the presence of TssDNA at 1:5 (blue). FUS, Fused in sarcoma; HSQC, Heteronuclear single quantum coherence spectroscopy; NTD, N-terminal domain; ssDNA, single-stranded DNA; TssDNA, telomeric ssDNA. (TIF) [file pbio.3000327.s013.tif]
